# MEIAO: A Multi-Strategy Enhanced Information Acquisition Optimizer for Global Optimization and UAV Path Planning

**DOI:** 10.3390/biomimetics10110765

**Published:** 2025-11-12

**Authors:** Yongzheng Chen, Ruibo Sun, Jun Zheng, Yuanyuan Shao, Haoxiang Zhou

**Affiliations:** 1 School of Mathematics, University of Edinburgh, Edinburgh EH8 8FH, UK; s2837889@ed.ac.uk; 2 School of Engineering, University of California, Merced, CA 95344, USA; ruibosun@ucmerced.edu; 3 Taizhou Institute, Zhejiang University, Taizhou 318000, China; shaoyuanyuan@tzizju.cn

**Keywords:** information acquisition optimizer, UAV path planning, differential evolution operator, metaheuristic algorithm, global optimization

## Abstract

With the expansion of unmanned aerial vehicles (UAVs) into complex three-dimensional (3D) terrains for reconnaissance, rescue, and related missions, traditional path planning methods struggle to meet multi-constraint and multi-objective requirements. Existing swarm intelligence algorithms, limited by the “no free lunch” theorem, also face challenges when the standard Information Acquisition Optimizer (IAO) is applied to such tasks, including low exploration efficiency in high-dimensional search spaces, rapid loss of population diversity, and improper boundary handling. To address these issues, this study proposes a Multi-Strategy Enhanced Information Acquisition Optimizer (MEIAO). First, a Levy Flight-based information collection strategy is introduced to leverage its combination of short-range local searches and long-distance jumps, thereby broadening global exploration. Second, an adaptive differential evolution operator is designed to dynamically balance exploration and exploitation via a variable mutation factor, while crossover and greedy selection mechanisms help maintain population diversity. Third, a globally guided boundary handling strategy adjusts out-of-bound dimensions to feasible regions, preventing the generation of low-quality paths. Performance was evaluated on the CEC2017 (dim = 30/50/100) and CEC2022 (dim = 10/20) benchmark suites by comparing MEIAO with eight algorithms, including VPPSO and IAO. Based on the mean, standard deviation, Friedman mean rank, and Wilcoxon rank-sum tests, MEIAO demonstrated superior performance in local exploitation of unimodal functions, global exploration of multimodal functions, and complex adaptation on composite functions while exhibiting stronger robustness. Finally, MEIAO was applied to 3D mountainous UAV path planning, where a cost model considering path length, altitude standard deviation, and turning smoothness was established. The experimental results show that MEIAO achieved an average path cost of 253.9190, a 25.7% reduction compared to IAO (341.9324), with the lowest standard deviation (60.6960) among all algorithms. The generated paths were smoother, collision-free, and achieved faster convergence, offering an efficient and reliable solution for UAV operations in complex environments.

## 1. Introduction

With the rapid advancement of unmanned aerial vehicle (UAV) technology in reconnaissance, civilian rescue, environmental monitoring, and power inspection, the operational scenarios of UAVs have evolved from simple open-air spaces to complex three-dimensional (3D) environments, such as mountainous areas with multiple obstacles and densely built urban regions. This shift has significantly increased the complexity of UAV path planning tasks [1,2,3]. In practical operations, UAV path planning must not only ensure that flight routes avoid terrain and obstacles to guarantee flight safety but also balance multiple competing objectives. For example, in reconnaissance missions, the path should be as short as possible to minimize exposure time while maintaining stable altitude variations to reduce the probability of radar detection [4,5]. In contrast, during mountain rescue missions, the path should maintain high smoothness to ensure the stable operation of onboard payloads (e.g., infrared life detectors or material delivery systems), avoiding frequent turns or abrupt altitude changes that could cause equipment failure or mission interruption. These multi-constraint and multi-objective requirements make traditional path planning methods—those based on manual experience or simple geometric calculations—increasingly inadequate to meet modern UAV demands for precision, safety, and efficiency. Consequently, there is an urgent need for more efficient intelligent optimization algorithms to provide robust technical support for UAV path planning [6,7,8].

However, UAV path planning in complex environments still faces several pressing challenges that remain to be addressed. First, the complexity of environmental modeling and constraint handling poses a significant difficulty. In three-dimensional scenarios, the terrain and obstacles often exhibit irregular and nonuniform distributions, requiring precise mathematical modeling to accurately represent mountainous and obstacle-rich environments. During the search for feasible paths, algorithms must strictly satisfy the hard constraint that “the path must not penetrate any obstacle region,” while also avoiding excessive confinement to local feasible domains that may lead to missing the global optimum [9,10]. Second, achieving a proper balance among multiple optimization objectives remains a core challenge. Objectives such as path length, altitude standard deviation, and turning smoothness are inherently coupled and often conflicting. For instance, excessive pursuit of shorter path length may cause the route to traverse steep terrains, resulting in abrupt altitude changes; conversely, overemphasizing smoothness may lead to overly detoured paths, increasing both energy consumption and flight time. Traditional optimization algorithms often struggle to strike an optimal balance among these competing objectives, leading to the classic “trade-off dilemma.” Finally, there exists a tension between algorithmic robustness and real-time performance. The high-dimensional search spaces corresponding to complex 3D environments tend to slow down the convergence of certain optimization algorithms, thereby compromising their reliability and making it difficult to ensure stable and timely path support for UAV operations [11,12].

In this context, swarm intelligence algorithms, as an important branch of metaheuristic optimization, have gradually become an ideal technical approach to solving UAV path planning challenges due to their ability to simulate the cooperative behaviors of biological populations [13,14]. Compared with traditional optimization methods, swarm intelligence algorithms offer distinct advantages. First, they demonstrate strong environmental adaptability. These algorithms do not rely on gradient information or explicit analytical models but instead explore complex constrained spaces autonomously through iterative searches and information exchange among individuals. For example, in the mountainous obstacle environments constructed in this study, swarm intelligence algorithms can dynamically update the positions of individuals to automatically avoid obstacle regions and identify feasible paths. Second, they exhibit flexible multi-objective optimization capabilities. By properly setting weighting coefficients or employing multi-objective optimization strategies, swarm intelligence algorithms can effectively balance multiple objectives in UAV path planning. Third, they show excellent scalability. By integrating targeted enhancement strategies, these algorithms can flexibly adjust their exploration and exploitation abilities to suit path planning scenarios of varying complexity. For instance, introducing specialized search mechanisms can enhance global exploration, while designing adaptive parameter adjustment strategies can improve local exploitation accuracy, thereby enabling swarm intelligence algorithms to better address the requirements of UAV path planning in complex environments [15,16,17].

In recent years, scholars at home and abroad have conducted extensive research on the improvement and application of swarm intelligence algorithms, resulting in a rich and diverse system of optimization techniques. For instance, Eberhart and Kennedy proposed the Particle Swarm Optimization (PSO) algorithm inspired by the foraging behavior of bird flocks [18], and N. Metropolis et al. introduced the Simulated Annealing (SA) algorithm inspired by the annealing process in metallurgy [19]. The Grey Wolf Optimizer (GWO) was designed by Mirjalili et al. based on the social hierarchy and hunting strategies of grey wolves [20], and the Whale Optimization Algorithm (WOA) was proposed to simulate the hunting behavior of humpback whales, including search, encircling, and spiral updating phases [21], Similarly, the Harris Hawks Optimization (HHO) algorithm was inspired by the cooperative hunting process of Harris’s hawks [22]; the Dung Beetle Optimizer (DBO) was developed based on the rolling, dancing, foraging, stealing, and breeding behaviors of dung beetles [23]; and Fu et al. proposed the Secretary Bird Optimization Algorithm (SBOA) inspired by the survival strategies of secretary birds [24].

In addition, the Red-billed Blue Magpie Optimizer (RBMO) was designed by simulating the search, pursuit, attack, and food storage behaviors of red-billed blue magpies [25], while the Arithmetic Optimization Algorithm (AOA) utilized the basic mathematical operations of addition, subtraction, multiplication, and division—where the latter two introduce strong dispersion characteristics—to guide the search process [26]. The Great Wall Construction Algorithm (GWCA) was inspired by the competitive and elimination mechanisms observed among workers during the construction of the Great Wall in ancient China [27]. Moreover, the Cuckoo Catfish Optimizer (CCO) simulated the searching, predation, and parasitic behaviors of cuckoo catfish observed in nature [28]; the Philoponella Prominens Optimizer (PPO) modeled the unique mating, post-copulatory escape, sexual cannibalism, and predation behaviors of the spider Philoponella prominens [29]; and finally, the Kirchhoff’s Law Algorithm (KLA) was developed as a novel optimization method inspired by electrical circuit principles, particularly Kirchhoff’s Current Law (KCL) [30]. Inspired by the story of Tian Ji’s horse racing in Chinese history, which tells how Tian Ji used his strengths to offset his opponent’s weaknesses and ultimately win the race, the Tianji’s Horse Racing Optimization (TSRO) [31] was proposed. Additionally, based on the observation of the tornado lifecycle and how thunderstorms and storms utilize the Coriolis force to evolve into tornadoes, the Tornado Optimizer with Coriolis Force (TOCF) [32] was proposed. This optimization method directly simulates the evolution of storms into tornadoes by applying the Coriolis force.

However, stochastic metaheuristic algorithms also possess inherent limitations. In 1997, David H. Wolpert and William G. Macready [33] proposed the “No Free Lunch” (NFL) theorem, which logically demonstrates that no single metaheuristic algorithm can optimally solve all optimization problems. In other words, while a given intelligent algorithm may perform well on certain problems, it may yield unsatisfactory results on others. To address this issue, researchers have increasingly explored hybrid approaches that integrate multiple intelligent algorithms to achieve superior performance in UAV path planning [34,35,36,37]. For example, Fu et al. [25], inspired by the foraging, chasing, attacking, and food-storing behaviors of red-billed blue magpies, proposed the Red-Billed Blue Magpie Optimizer (RBMO) for two-dimensional and three-dimensional UAV path planning. Huang et al. [38] developed a particle swarm optimization algorithm based on cylindrical vectors combined with waveform and S-shaped functions. Qu et al. [39] proposed a novel hybrid Grey Wolf Optimizer (HGWO) by integrating the Simplified Grey Wolf Optimizer (SGWO) with an improved Symbiotic Organisms Search (MSOS). Similarly, Yu et al. [40] designed a Hybrid Grey Wolf Differential Evolution (HGWODE) algorithm. These studies collectively demonstrate that hybrid intelligent algorithm frameworks can effectively overcome the limitations of individual metaheuristic methods, thereby enhancing the efficiency, adaptability, and robustness of UAV path planning in complex environments.

Although swarm intelligence algorithms have application value in many fields, their limitations remain quite evident. First, there are shortcomings in search performance—they are prone to falling into local optima due to positive feedback mechanisms, and their search efficiency declines significantly in high-dimensional problems [41]. Second, there is a gap between theory and practice—most algorithms lack rigorous convergence proofs, their performance relies on empirical parameter tuning, and they exhibit “black-box” characteristics. Finally, there are issues with application barriers and costs—core parameters greatly influence the results but lack clear setting standards, while large-scale population computations increase time and hardware costs, limiting their implementation in certain scenarios [42].

The Information Acquisition Optimizer (IAO), proposed in recent years as a novel swarm intelligence algorithm, is designed to simulate the human information processing cycle of “collection–evaluation–analysis and organization”. Through iterative updates, IAO has demonstrated potential in solving low- to medium-dimensional numerical optimization problems [43]. However, when applied to complex tasks such as UAV path planning, its inherent limitations become increasingly apparent, mainly in three aspects. First, during the information collection phase, the standard IAO updates an information agent’s position by combining the difference between two randomly selected agents with a random factor. In the high-dimensional search spaces typical of UAV path planning, this mechanism can induce excessive randomness in search directions, restrict the global search scope, and cause the algorithm to become trapped in local optima. Second, in the analysis and organization phase, standard IAO overly relies on the best information agent in the current iteration and lacks a dynamic parameter adjustment mechanism. This leads to rapid convergence of the population, a quick loss of diversity, and insufficient generation of diverse path solutions in the middle-to-late iterations. As a result, UAVs cannot obtain alternative paths, and the algorithm struggles to escape previously trapped local optima. Third, in boundary handling, standard IAO uses a simple truncation strategy, directly setting any out-of-bound dimension to the boundary value. This approach tends to cause out-of-bound agents to cluster near obstacle edges or search space boundaries, wasting computational resources on low-quality path searches and reducing the feasibility of the generated paths. For example, if the UAV’s altitude dimension exceeds the boundary and is forcibly set to the boundary height, collisions with surrounding terrain or obstacles may occur, compromising flight safety. These shortcomings severely limit the effectiveness of IAO in UAV path planning, highlighting the urgent need for targeted improvement strategies to comprehensively enhance its performance.

Based on the above research background and existing challenges, this study aims to enhance the accuracy, robustness, and real-time performance of UAV path planning. Building on the standard IAO algorithm, multiple improvement strategies are integrated to propose the Multi-Strategy Enhanced Information Acquisition Optimizer (MEIAO), with comprehensive research conducted on its design, validation, and application. First, to address the low exploration efficiency during the IAO information collection phase, MEIAO introduces a Levy Flight-based information collection strategy. Leveraging the “short-range local search with occasional long-distance jumps” characteristic of Levy flights, this strategy expands the algorithm’s global search scope and strengthens its ability to escape local optima. Second, to tackle the rapid loss of diversity during the IAO information analysis phase, MEIAO incorporates an adaptive differential evolution operator. By employing a mutation factor that dynamically changes with iteration number, MEIAO enhances exploration in early iterations with a larger mutation factor and focuses on exploitation in later iterations with a smaller factor. Combined with binomial crossover and greedy selection, this mechanism maintains population diversity while improving convergence accuracy. Finally, to remedy IAO’s inadequate boundary handling, MEIAO adopts a globally guided boundary handling strategy, adjusting out-of-bound dimensions to reasonable regions between the boundary and the global best solution, thereby improving path quality.

The main contributions of this work are as follows:

(1) The integration of Levy Flight-based information collection, adaptive differential evolution, and globally guided boundary handling into the original IAO results in the proposed MEIAO, which comprehensively enhances algorithm performance in high-dimensional, multimodal, and complex-constrained scenarios.
(2) Extensive experiments on the CEC2017 and CEC2022 benchmark suites compare MEIAO with eight mainstream algorithms. Evaluation metrics including mean, standard deviation, Friedman mean rank, and Wilcoxon rank-sum tests demonstrate MEIAO’s superior performance in local exploitation of unimodal functions, global exploration of multimodal functions, and complex adaptation of composite functions.
(3) MEIAO is applied to UAV path planning in 3D mountainous environments, with a comprehensive cost optimization model considering path length, altitude standard deviation, and turning smoothness. Through quantitative analysis of path costs (best/worst/average), 3D path visualization, and convergence curves, MEIAO is shown to generate shorter, smoother, and collision-free feasible paths. It also exhibits strong robustness across multiple trials, providing an efficient and reliable path optimization solution for practical UAV operations.

The subsequent chapters of this paper are organized as follows: Section 2 provides a detailed description of the basic principles of the IAO algorithm, including the mathematical models and iterative processes for its three stages—information collection, evaluation, and analysis/organization. This chapter also thoroughly presents the specific implementation and mathematical derivation of the three improvement strategies integrated into MEIAO. Section 3 conducts numerical experiments, quantitatively comparing the optimization accuracy, convergence speed, and robustness of MEIAO with benchmark algorithms based on the CEC test suites, and performs ablation studies to analyze the individual contributions and synergistic effects of each improvement strategy. Section 4 focuses on the application of UAV path planning, constructing a complete optimization framework and validating through simulations MEIAO’s capability to generate high-quality paths in complex three-dimensional environments. Finally, Section 5 summarizes the research findings, outlines MEIAO’s performance advantages and limitations, and discusses future directions for algorithmic improvements and application expansions.

## 2. Information Acquisition Optimizer and the Proposed Methodology

### 2.1. Information Acquisition Optimizer (IAO)

#### 2.1.1. Information Collection

Information collection is a critical step for acquiring useful data. To ensure a more comprehensive gathering of initial information, individuals employ multiple methods to collect information from diverse sources, forming an initial information system. This process can be expressed as follows [43]:

(1) $$\begin{array}{c} x_{i}^{iter+1}=x_{i}^{iter}+\vartheta \;\times \;\left(x_{i}^{r1}-x_{i}^{r2}\right) \end{array}$$

where iter denotes the current iteration, $x_{i}^{iter}$ represents the state of the $i^{th}$ information entity at the ${iter}^{th}$ iteration, $x_{i}^{iter+1}$ is the updated information entity after the initial information collection, $x_{i}^{r1}$ and $x_{i}^{r2}$ are two randomly generated information entities at the iter iteration, and ϑ is a random number in the interval [0, 1], representing the factors influencing the information collection process.

#### 2.1.2. Information Filtering and Evaluation

In today’s environment of information overload, the processes of information filtering and evaluation have become critical mechanisms for individuals to rapidly identify relevant and useful information. These processes not only effectively eliminate inaccurate or misleading information but also significantly enhance the overall quality of the acquired information. This mechanism provides inspiration for the mathematical representation of the exploration phase in the IAO algorithm, which can be expressed by Equation (2) [43]:

(2) $$\begin{array}{c} x_{i}^{iter+1}=\left\{\begin{array}{c} x_{i}^{iter}-∆\;\times \;rand\;\times \;\left(x_{i}^{rand}-x_{i}^{iter}\right),\;\;\;if\;rand<0.5\\ x_{i}^{iter}+∆\;\times \;rand\;\times \;\left(x_{i}^{rand}-x_{i}^{iter}\right),\;\;\;if\;rand\geq 0.5 \end{array}\right. \end{array}$$

where rand is a randomly generated number in the interval [0, 1], and ∆ represents errors arising from subjective factors during the information filtering and evaluation process, as defined in Equation (3).

(3) ∆=cosπ2×|Γ|ΞΞ=2×mod3.468×v×1−β×a cosγ×104,1

Ξ denotes the subjective influence factor, serving as a quantitative measure of individual subjectivity and playing a crucial role. It reflects how personal preferences, experience, emotions, and preconceived notions may lead to overly optimistic or pessimistic judgments, thereby affecting the final acquisition and application of information. Moreover, due to cognitive limitations and contextual influences, Ξ also represents potential errors in information processing, which may occur randomly or result from systematic biases such as confirmation bias or groupthink. The randomness of Ξ further highlights the inherent uncertainty in the information filtering and evaluation process, implying that even when faced with the same information, different individuals—or the same individual at different times (due to changes in subjective state)—may reach different evaluation outcomes [43]. Both β and γ are random numbers generated within the interval [0, 1].

Γ is defined as the reliability factor, and its mathematical model is inspired by humans’ ability to dynamically adjust during the information filtering and evaluation process. As information transitions from complex to simple, the processing accordingly becomes simpler. Thus, Γ represents the algorithm’s capability to self-adjust based on information quality at different iteration stages to optimize its behavior. This design enhances the algorithm’s adaptability and flexibility, significantly improving the reliability and effectiveness of the results. The value of Γ is calculated using Equation (4), where Max_iter denotes the maximum number of iterations. The mathematical model of Γ consists of three components: a sine function term, a logarithmic function term, and an information quality factor Φ. The exponential treatment of the sine function provides a dynamic adjustment mechanism, exhibiting significant variation in the early iterations to facilitate rapid strategy adaptation, while its rate of change slows in later iterations, supporting algorithmic stability and fine-tuning. The logarithmic component ensures stability throughout the iteration process, whereas the information quality factor Φ guarantees that information quality remains a critical influence on Γ, preventing the algorithm from over-optimizing iteration dynamics at the expense of fundamental information quality requirements. As the number of iterations increases, this influence gradually smooths, simulating the transition in information processing from coarse filtering to fine filtering [43].

(4) $$\begin{array}{c} \Gamma =sin\left({\left(\frac{\pi }{4}\right)}^{\frac{iter}{Max\_iter}}\right)+\Phi +\frac{\log_{10}\frac{iter}{Max\_iter}}{8} \end{array}$$

Its value is calculated using the following formula, where δ is a random number generated within the interval [0, 1]. Φ can be regarded as a function of the iteration number iter, and it influences the quality of information acquisition by adjusting the phase and amplitude of a cosine function. During the iteration process, this quality factor Φ affects the reliability factor Γ, which in turn impacts the state $x_{i}^{iter+1}$ of individual i. In this way, the IAO algorithm can simulate the adaptive adjustment behavior of individuals when facing different information, thereby achieving optimal information acquisition.

(5) $$\begin{array}{c} \Phi =sin\left(2\times \delta +1\right)\times \left(1-\frac{iter}{Max\_iter}\right) \end{array}$$

#### 2.1.3. Information Analysis and Organization

The purpose of information analysis and organization is to identify useful information from the filtered data and to transform convertible information identified in the previous stage into valuable knowledge, thereby increasing the probability of acquiring optimal information entities. In the development phase of the IAO algorithm, this process is mathematically represented as in [43]:

(6) $$\begin{array}{c} x_{i}^{iter+1}=\left\{\begin{array}{c} x_{i}^{best}\;\times \;cos\left(\frac{\pi }{2}\;\times \;\sqrt{\Lambda^{1/3}}\right)-\varepsilon \;\times \;\left(\frac{1}{D}\sum_{i=1}^{d}x_{i}^{best}-x_{i}^{best}\right),\;\;\;\;\;\;\;\;\;\;\;\;\;\;\;\;\;\;\;\;\;\;\;\;\;\;\;\;\;\;\;\;\;\;\;\;\;\;\;\;\;\;\;\;\;\;\;\;\;\;\;\;\;\;\;\;\;if\;\Phi <0.5\\ x_{i}^{best}\;\times \;cos\left(\frac{\pi }{2}\;\times \;\sqrt{\Lambda^{1/3}}\right)-0.8\;\times \;\left(\xi \;\times \;\kappa \;\times \;\frac{1}{D}\sum_{i=1}^{d}x_{i}^{best}-\left(2\;\times \;\omega -1\right)\;\times \;x_{i}^{best}\right),\;\;\;\;\;\;\;\;\;otherwise \end{array}\right. \end{array}$$

where $x_{i}^{best}$ denotes the optimal information entity generated in the previous iteration, $\frac{1}{D}\sum_{i=1}^{d}x_{i}^{best}$ represents the average of the optimal information entities from the previous iteration, and ε,ξ,κ,ω are random numbers in the interval [0, 1]. Λ is a control factor for the information analysis and organization process, defined as follows:

(7) $$\begin{array}{c} \Lambda =2^{\left(\sqrt{\left|\Gamma \right|}-2\right)} \end{array}$$

The effectiveness of information analysis depends on an individual’s ability to tailor their approach based on how reliable the information is. This dynamic optimization of focus and detail directly leads to more accurate and useful results. This is formally represented by Lambda Λ, a parameter controlled by the reliability factor, Gamma $\left(\Gamma \right)$. Essentially, Λ determines how rigorously and meticulously information is handled. If the information is trustworthy (a high Γ), Λ increases to support deeper investigation and wider correlation. If the information is suspect (a low Γ), Λ decreases, shifting the process to a more cautious mode to minimize the influence of potentially erroneous data.

### 2.2. Proposed Multi-Strategy Enhanced Information Acquisition Optimizer (MEIAO)

#### 2.2.1. Levy Flight-Based Information Collection Strategy

In the standard IAO algorithm’s information collection phase, the core mechanism updates the current information entity’s position by multiplying the difference between two randomly selected information entities with a random factor ϑ. While this approach enables diverse initial solution generation, it often suffers from low exploration efficiency and excessive randomness in search direction within high-dimensional, complex search spaces, leading to slow convergence or entrapment in local optima. To address this limitation, the MEIAO algorithm introduces a Levy Flight mechanism to enhance the information collection strategy. By leveraging the dynamic balance of long-distance jumps and short-distance searches inherent in Levy Flights, the algorithm’s global exploration capability in complex search spaces is significantly improved.

**Figure 1:**
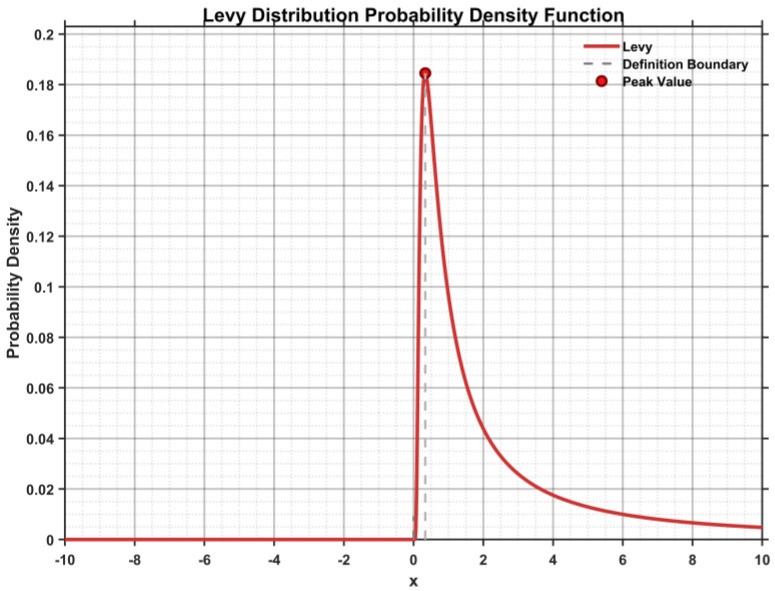
Levy distribution probability density function diagram.

As shown in Figure 1, the probability density function of Levy Flights follows a power-law distribution, with step-length characteristics such that most of the time the search is local and short-range, while occasional long-distance jumps occur. This behavior closely resembles natural phenomena such as animal foraging and bird migration, effectively preventing the algorithm from stagnating in local regions. In the MEIAO algorithm, the Levy Flight step length is generated using Equation (8):

(8) $$\begin{array}{c} z=\frac{u}{{\left\|v\right\|}^{1/\beta }} \end{array}$$

where β is the characteristic exponent of the Levy Flight, ranging from 1 to 2. In this study, β=1.5 is selected to balance global exploration and local exploitation. v and u are independent random variables drawn from standard normal distributions, i.e., $u\sim N\left(0,\sigma_{u}^{2}\right)$, $v\sim N\left(0,1\right)$; and $\sigma_{u}$ is the standard deviation of u, calculated as follows:

(9) $$\begin{array}{c} \sigma_{u}={\left(\frac{\Gamma \left(1+\beta \right)sin\left(\frac{\pi \beta }{2}\right)}{\Gamma \left(\frac{\left(1+\beta \right)}{2}\right)\beta 2^{\frac{\left(\beta -1\right)}{2}}}\right)}^{1/\beta } \end{array}$$

where $\Gamma \left(\cdot \right)$ is the Gamma function, ensuring the rationality and stability of the Levy Flight step-length distribution.

Integrating the Levy Flight step length into the information collection position update, the improved update formula becomes:

(10) $$\begin{array}{c} x_{i}^{iter+1}=x_{i}^{iter}+\vartheta \;\times \;{RL}_{i}\;\times \;\left(x_{i}^{r1}-x_{i}^{r2}\right) \end{array}$$

where ${RL}_{i}$ is the Levy Flight step-length vector corresponding to the $i^{th}$ information entity; $x_{i}^{r1}$ and $x_{i}^{r2}$ are two randomly selected, distinct information entities; and ϑ is a random factor in the interval [−1, 1], controlling the influence of the step length. By incorporating Levy Flight steps, the algorithm can dynamically adjust the exploration range during the information collection phase: when the search region is far from the optimal solution, larger Levy steps enable long-distance jumps to rapidly expand the search boundary; when near the optimal region, smaller steps focus on local search, providing high-quality initial solutions for subsequent information filtering and evaluation stages.

#### 2.2.2. Adaptive Differential Evolution Operator

**Figure 2:**
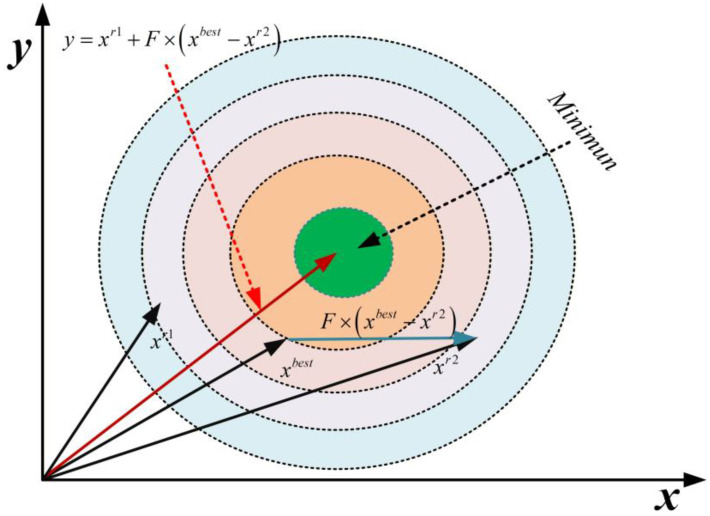
The schematic diagram of the Differential Evolution strategy operation.

In the standard IAO algorithm’s information analysis and organization phase, position updates primarily rely on a combination of the optimal information entity and random factors. While this approach enables local exploitation, it often suffers from rapid loss of population diversity and stagnation in later-stage convergence when addressing multimodal, high-dimensional optimization problems. To enhance the algorithm’s adaptability to complex problems, the MEIAO algorithm introduces an adaptive differential evolution (DE) operator, as illustrated in Figure 2. Through mutation, crossover, and greedy selection operations, the operator dynamically optimizes the population structure, maintaining diversity while improving convergence accuracy.

1. Design of the adaptive mutation factor

The mutation factor F is a core parameter of the differential evolution operator, directly influencing the balance between exploration and exploitation. In standard DE algorithms, F is often set as a fixed value, which fails to accommodate the differing optimization demands at various iteration stages: a larger F is required during early iterations to enhance exploration, while a smaller F is preferred in later iterations to focus on exploitation. In MEIAO, we adopt an adaptive mutation factor F that is updated dynamically as the algorithm proceeds; the specific update rule is defined in Equation (11).

(11) $$\begin{array}{c} F=F_{min}+\left(F_{max}-F_{min}\right)\times \left(1-\frac{iter}{Max\_iter}\right) \end{array}$$

where $F_{min}=0.2$, $F_{max}=0.6$ (the maximum value of the mutation factor), Max_iter denotes the maximum number of iterations, and iter is the current iteration. As the number of iterations increases, F decreases linearly from $F_{max}$ to $F_{min}$, enabling a dynamic transition from global exploration in the early stages to local exploitation in the later stages.

The mutation operation based on the adaptive mutation factor is given by Equation (12):

(12) $$\begin{array}{c} \begin{array}{c} y=x_{i}^{r1}+F\;\times \;\left(x_{i}^{best}-x_{i}^{r2}\right) \end{array} \end{array}$$

where y is the intermediate vector generated after mutation, F is the adaptive mutation factor, $x_{i}^{best}$ represents the current global optimal information entity, and $\left(x_{i}^{best}-x_{i}^{r2}\right)$ denotes the difference between the current position and the global optimum. By guiding the mutation direction using the global best information entity, the algorithm can quickly converge toward the optimal region, avoiding directionless random searches and improving the efficiency of the mutation operation.

2. Crossover operation and greedy selection

The purpose of the crossover operation is to generate new individuals through recombination, thereby maintaining population diversity. The MEIAO algorithm employs a binomial crossover strategy with a crossover probability of $p_{cr}=0.7$, and a randomly selected crossover dimension $j_{0}$ is set to ensure that each new individual inherits at least one dimension from the mutated vector, preventing the crossover operation from being ineffective. The crossover operation is defined as follows:

(13) $$\begin{array}{c} z\left(j\right)=\left\{\begin{array}{l} y\left(j\right),\;\;\;\;\;\;\;\;\;\;if\;j=j_{0}\;or\;rand\;\leq p_{cr}\\ x\left(i,j\right),\;\;\;\;\;\;\;\;\;\;\;\;\;\;\;\;\;\;\;\;\;\;\;\;\;\;\;\;\;\;\;\;\;\;\;\;\;else \end{array}\right. \end{array}$$

where z is the new individual generated after crossover, j is the dimension index, $x\left(i,j\right)$ is the $j^{th}$ dimension of the $i^{th}$ information entity, and rand is a random number in the interval [0, 1].

The greedy selection operation compares the fitness of the new individual z with that of the original individual $x_{i}$, retaining the better one, as expressed in Equation (14):

(14) $$\begin{array}{l} x_{i}^{iter+1}=\left\{\begin{array}{c} z,\;\;\;\;\;\;\;\;if\;f\left(z\right)<f\left(x_{i}^{iter}\right)\\ x_{i}^{iter},\;\;\;\;\;\;\;\;\;\;\;\;\;\;\;\;\;\;\;\;\;\;\;\;\;\;\;else \end{array}\right. \end{array}$$

where $f\left(\cdot \right)$ denotes the fitness function. Through greedy selection, the algorithm ensures gradual improvement in population quality while avoiding the interference of inferior individuals in the optimization process.

#### 2.2.3. Global Optimal Guided Boundary Processing Strategy

In the standard IAO algorithm, boundary violations are handled using a simple truncation method, which directly sets any out-of-bounds dimension to its corresponding boundary value. This approach often causes out-of-bounds individuals to cluster near the boundaries, reducing population diversity and failing to utilize existing optimal information to guide the search direction.

**Figure 3:**
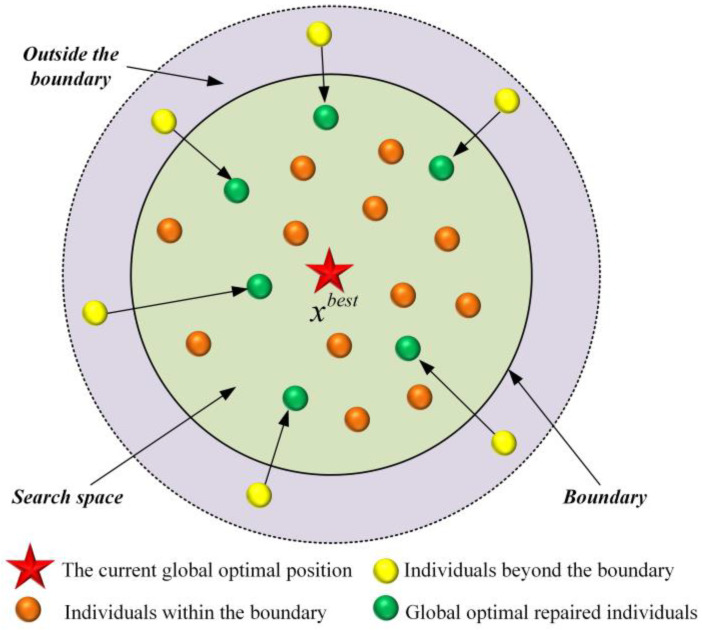
Global optimal guided boundary processing strategy diagram.

To address this issue, the MEIAO algorithm introduces a global-best-guided boundary handling strategy. As show in Figure 3, when an information entity exceeds the search bounds, its out-of-range dimensions are adjusted toward a reasonable region between the boundary and the global best solution. This approach not only ensures solution feasibility but also improves the quality of boundary-violating individuals.

The specific formulation of the global-best-guided boundary handling strategy is given as follows:

(15) $$\begin{array}{l} x_{i,j}=\left\{\begin{array}{l} x_{j}^{best}+0.5\;\times \;rand\;\times \;\left({ub}_{j}-x_{j}^{best}\right),if\;x_{i,j}>{ub}_{j}\\ x_{j}^{best}-0.5\;\times \;rand\;\times \;\left(x_{j}^{best}-{lb}_{j}\right),\;if\;x_{i,j}<{lb}_{j}\\ x_{i},\;\;\;\;\;\;\;\;\;\;\;\;\;\;\;\;\;\;\;\;\;\;\;\;\;\;\;\;\;\;\;\;\;\;\;\;\;\;\;\;\;\;\;\;\;\;\;\;\;\;\;\;\;\;\;\;\;\;elsewhise \end{array}\right. \end{array}$$

where $x_{i,j}$ is the $j^{th}$ dimension of the $i^{th}$ information entity, ${ub}_{j}$ and ${lb}_{j}$ are the upper and lower bounds of the $j^{th}$ dimension, $x_{j}^{best}$ is the $j^{th}$ dimension of the global best information entity, and rand is a random number in the interval [0, 1].

This strategy enhances the algorithm’s performance through two key advantages. First, the adjustment direction of out-of-bounds individuals is guided by the global best information entity, avoiding quality degradation caused by random adjustments and ensuring that boundary-violating individuals still have the potential to approach the optimal solution. Second, the incorporation of the random factor 0.5×rand ensures that adjusted out-of-bounds individuals maintain a certain level of diversity, preventing multiple individuals from clustering at the same position and preserving population diversity. This boundary-handling strategy is applied consistently across all stages of the algorithm—including information collection, filtering and evaluation, and analysis and organization—thereby safeguarding both solution quality and diversity throughout the optimization process.

Based on the above discussion, the pseudocode for MEIAO is presented in Algorithm 1.
**Algorithm** **1**. Pseudo-Code of MEIAO*1:  Initialize Problem Setting (population(N), dim, ub, lb), Max iterations*$\left(Max\_iter\right)$. *2:  Initialize the solution’s positions* ($x_{i}(i=1,2,\ldots N)$). *3:*  ***while*** iter=1:Max_iter*** do*** *4:     Calculate the energy factor*
A
*using Equation (8).* *5:    Generate a random number*
ϑ
*between*
[−1, 1]. *6:    Generate randomly two integers*
r1
*and*
r2
*between*
[1, N]. *7:    Generate a candidate population by Equations (8)–(10).* *8:*     ***for*** i=1:N*** do*** *9:          Calculate the fitness*
$x_{i}^{iter+1}$
*by Equations (2)–(5)*. *10:          Boundary processing by Equation (15).* *11:*    ***end for*** *12:*    ***for*** i=1:N*** do***
*13:         Calculate the fitness*
$x_{i}^{iter+1}$
*by Equations (6) and (7).* *14:        Boundary processing by Equation (15).* *15:    *
***end for*** *16:     Adaptive differential evolution operator by Equations (11)–(14).* *17:     Boundary processing by Equation (15).* *18:    **end for*** *19:    Update the position of solution and its fitness value.* *20:    Find the best solution position and fitness value so far.* *21:  **end while*** *22:  Return the best solution.*

### 2.3. Evaluation of MEIAO’s Computational Efficiency

In the design of algorithms, evaluating performance is crucial, with time complexity being a fundamental metric. For numerous optimization tasks, an algorithm must not only demonstrate strong problem-solving abilities but also satisfy constraints related to real-time execution. Time complexity indicates how the running time of an algorithm scales with increasing input size. Assessing the time complexity of an optimization method allows for estimating its computational demands when applied to large datasets. In the conventional IAO framework, the overall computational cost is mainly influenced by two components: the initialization of candidate solutions and the primary operations of the algorithm, which include computing the fitness of solutions and updating them. Denoting the population size as N, the maximum number of iterations as Max_iter, and the problem dimensionality as D, the initialization step has a computational cost of O(N). The computational complexity of the solution update process is O(Max_iter×N)+O(Max_iter×N×D), which encompasses the search for optimal positions and the update of all solution positions. Therefore, the total computational complexity of the IAO algorithm can be summarized as O(N×(Max_iter×D+1)). In MEIAO, our improvements do not introduce new loops or function evaluations but optimize and refine the formulas based on the original framework. Thus, the complexity of MEIAO remains O(N×(Max_iter×D+1)).

## 3. Numerical Experiments

### 3.1. Settings of Key Algorithm Parameters

This section assesses the effectiveness of the proposed MEIAO algorithm by employing the rigorous numerical optimization benchmarks from CEC2017 and CEC2022, and contrasts its performance with that of several alternative algorithms. The comparison algorithms include: Velocity Pausing Particle Swarm Optimization (VPPSO) [44], Improved multi-strategy adaptive Grey Wolf Optimization (IAGWO) [45], Advanced Differential Evolution(ADE) [46], Dung Beetle Optimizer (DBO) [23], Crested Porcupine Optimizer (CPO) [47], Animated Oat Optimization (AOO) [48], Holistic Swarm Optimization (HSO) [49], and Information acquisition optimizer (IAO) [43]. Table 1 contains the configuration details of every algorithm.

### 3.2. Qualitative Analysis of MEIAO

#### 3.2.1. Analysis of the Population Diversity

Within the realm of optimization algorithms, population diversity measures the degree of variation among the members of a population, where each member typically represents a candidate solution [50,51]. A decrease in population diversity frequently causes the algorithm to converge too early on local optima, thereby restricting its capacity to comprehensively explore the entire search space. Conversely, maintaining a higher diversity level enables more extensive exploration and increases the likelihood of finding the global optimum. In this study, the diversity of the MEIAO algorithm is assessed using Equation (16) [24,52].

(16) $$I_{C}\left(t\right)=\sqrt{\sum_{i=1}^{N}\;\sum_{d=1}^{D}\;{\left(x_{id}\left(t\right)-c_{d}\left(t\right)\right)}^{2}},$$

where $I_{C}\left(t\right)$ represents the diversity of the population at iteration t, N is the population size, D is the dimensionality of the problem, and $x_{id}\left(t\right)$ is the value of the $i^{th}$ individual in the $d^{th}$ dimension. The factor $c_{d}\left(t\right)$, computed via Equation (17), captures the spread of all individuals relative to the population’s centroid.

(17) $$c_{d}\left(t\right)=\frac{1}{D}\sum_{i=1}^{N}\;x_{id}\left(t\right).$$

**Figure 4:**
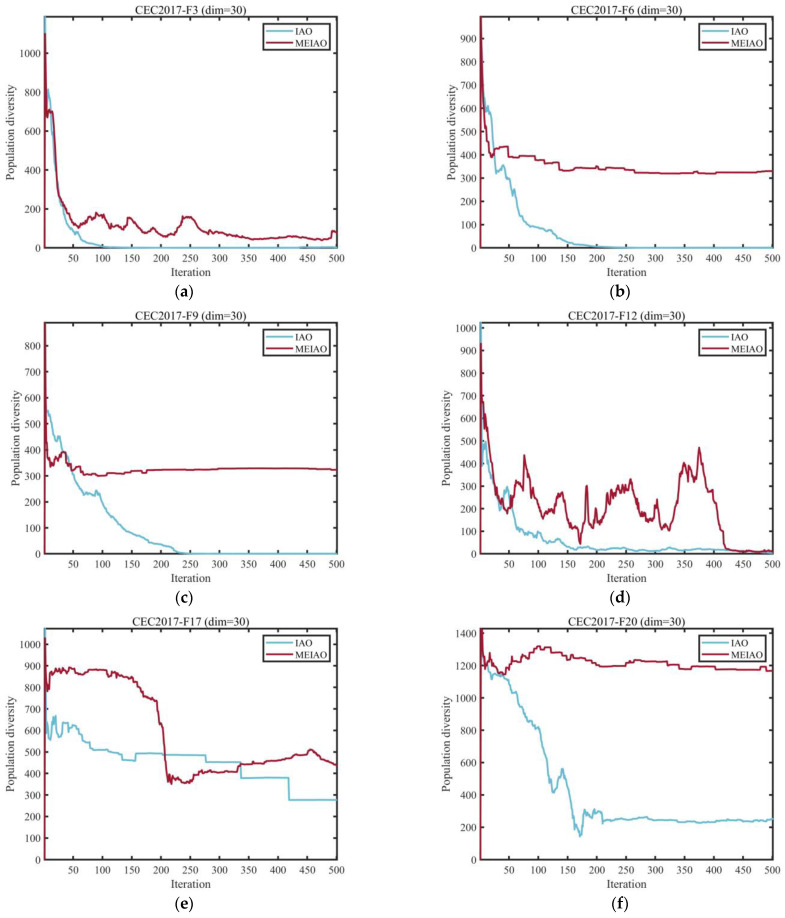

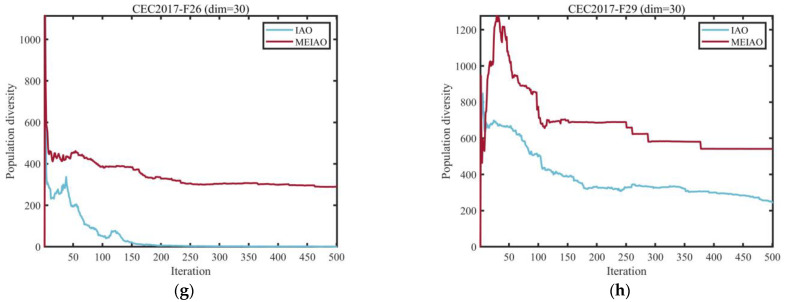
Assessment of population variation in MEIAO and IAO.

Figure 4 presents the population diversity evolution curves of MEIAO and the original IAO on representative functions from the CEC2017 test suite (dimension = 30). Diversity is calculated using Equation (16) and reflects the dispersion of individuals within the population, as well as the algorithm’s global exploration capability and resistance to premature convergence. The results indicate that MEIAO consistently maintains higher diversity across all test functions.

In the early iterations, MEIAO leverages the Levy Flight-based information collection strategy (S1) with power-law distributed step lengths to rapidly expand the search boundary, resulting in significantly higher diversity indices than IAO (e.g., for function F3, MEIAO maintains 800–900, while IAO only reaches 300–400). During the mid-iterations, its adaptive differential evolution operator (S2) dynamically adjusts the mutation factor, ensuring a smooth decline in diversity (e.g., F12 decreases from 700 to 400), thereby avoiding the sharp drop observed in IAO. In the later iterations, the global-best-guided boundary handling strategy (S3) preserves population differences through a random factor, allowing MEIAO to maintain moderate diversity (e.g., F26 stabilizes around 400), whereas IAO diversity approaches zero.

The superiority of MEIAO is particularly evident on multimodal functions (F9, F17) and composite functions (F26, F29), validating that the integration of multiple strategies effectively enhances the algorithm’s adaptability in complex search spaces.

#### 3.2.2. Evaluation of Exploration and Exploitation Behavior

The effectiveness of optimization algorithms relies on a fundamental dynamic between two competing objectives: global exploration and local refinement. Global exploration entails systematically probing diverse areas of the solution landscape to identify promising regions that might host the best overall solution. In contrast, local refinement concentrates on intensifying the search within the vicinity of known high-quality solutions, utilizing gathered information to enhance their accuracy [53,54].

Over-prioritizing exploration disperses computational effort inefficiently, as the algorithm may conduct widespread sampling without adequately refining potentially optimal zones, thus failing to capitalize on promising leads. Meanwhile, disproportionately focusing on refinement raises the probability of early convergence to suboptimal solutions, constraining the discovery of superior alternatives in unexplored domains. Consequently, maintaining an effective equilibrium between these competing processes is vital for achieving robust algorithmic performance. This section analyzes the exploration-exploitation characteristics of the MEIAO methodology, quantified through Equations (18) and (19) [24].

(18) $$Exploration\left(\%\right)=\frac{Div\left(t\right)}{Div_{max}}\times 100\%,$$

(19) $$Exploitation\left(\%\right)=\frac{\left|Div\left(t\right)-Div_{max}\right|}{Div_{max}}\times 100\%,$$

where $Div\left(t\right)$ represents the diversity metric at iteration t, computed according to Equation (20), while $Div_{max}$ indicates the highest diversity value observed across all iterations.

(20) $$Div\left(t\right)=\frac{1}{D}\sum_{d=1}^{D}\frac{1}{N}\sum_{i=1}^{N}|median\left(x_{d}\left(t\right)\right)-x_{id}\left(t\right)|.$$

**Figure 5:**
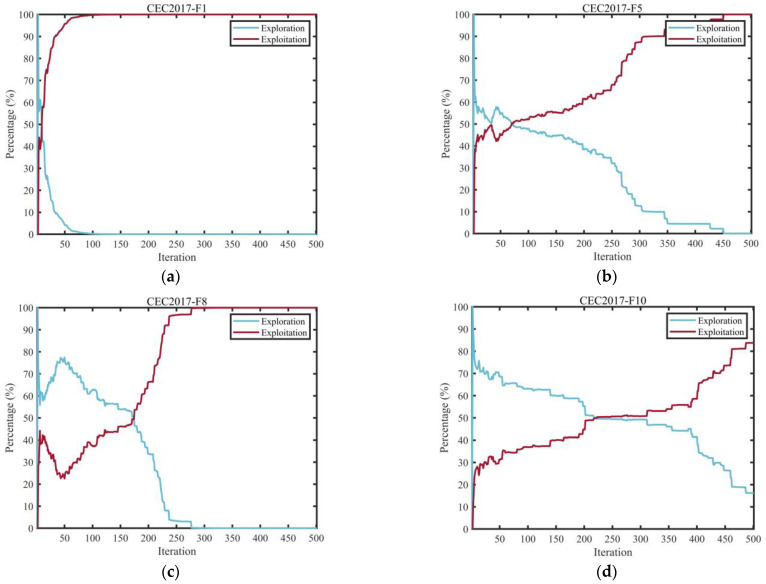

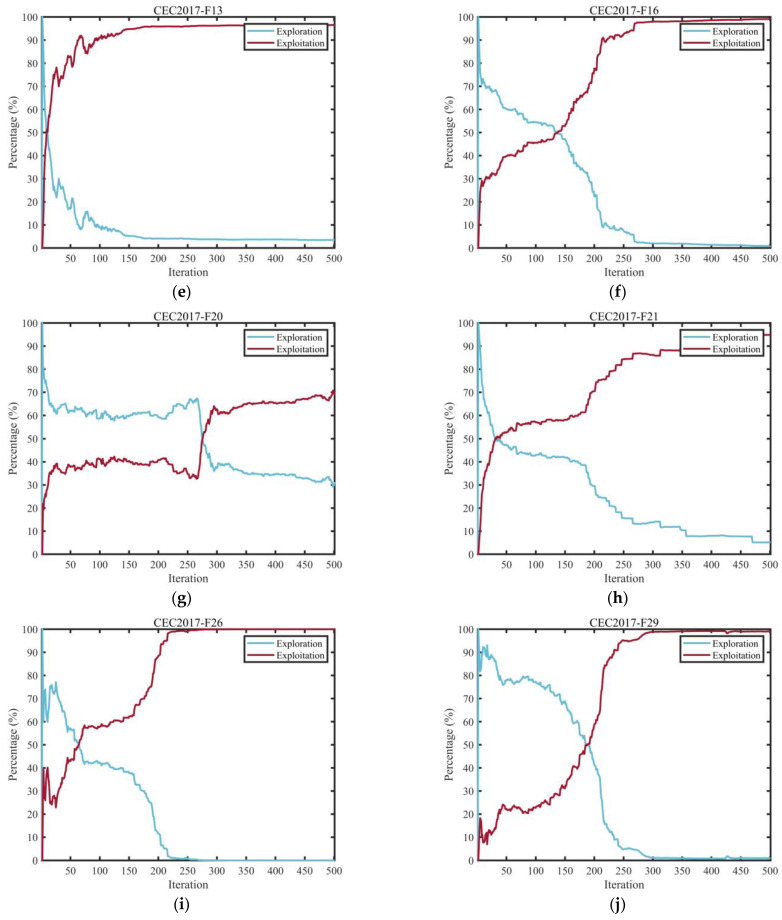
The analysis of the exploration and exploitation of MEIAO.

Figure 5 depicts the changing patterns of exploration and exploitation for the MEIAO algorithm across 10 selected benchmark functions (F1, F5, F8, F10, F13, F16, F20, F21, F26, F29) from the CEC2017 test suite, with each function evaluated in 30-dimensional space. The exploration and exploitation rates are calculated using Equations (18) and (19), respectively—where the exploration rate is represented by the ratio of current iteration diversity to maximum diversity, reflecting the global search coverage, and the exploitation rate is represented by the proportion of the difference between the two, indicating local fine-tuning capability. These metrics capture the algorithm’s ability to balance global traversal and local search.

From the evolution trends, MEIAO exhibits a reasonable dynamic pattern of “early-stage exploration, mid-stage balanced exploration and exploitation, late-stage exploitation.” In the early iterations (1–100), the exploration rate remains high at 70–90% (e.g., F1 exceeds 80%), benefiting from the long-distance jump characteristics of the Levy Flight strategy, which rapidly covers the search space to locate potential high-quality regions and lays the foundation for subsequent exploitation. During mid-iterations (100–300), the exploration rate gradually declines to 30–50%, while the exploitation rate rises to 50–70% (e.g., F10 shows an approximate 1:1 balance). The adaptive differential evolution operator dynamically adjusts the mutation factor F (linearly decreasing from 0.6 to 0.2), guiding the algorithm toward optimal regions while maintaining population diversity, thereby avoiding resource waste caused by excessive exploration. In the later iterations (300–500), the exploitation rate exceeds 80% (e.g., F26 exceeds 90%), with the algorithm leveraging the global-best-guided boundary handling strategy to perform fine-grained local optimization for improved solution accuracy, while the exploration rate remains at 10–20% to prevent premature convergence to local optima.

Moreover, MEIAO’s exploration-exploitation balance demonstrates strong adaptability across different types of test functions. For unimodal functions (F1, F5), the late-stage exploitation rate increases rapidly to efficiently converge to the global optimum. For multimodal functions (F13, F20), mid-stage exploration remains relatively high (~40%) to escape local optima. For composite functions (F26, F29), flexible switching between exploration and exploitation effectively addresses the complex search space structures. This dynamic balancing mechanism effectively overcomes the common shortcomings of traditional algorithms—such as insufficient exploration leading to local entrapment or insufficient exploitation hindering convergence—providing a key foundation for MEIAO’s superior performance across various optimization problems.

#### 3.2.3. Assessment of the Effects of the Proposed Strategy

**Figure 6:**
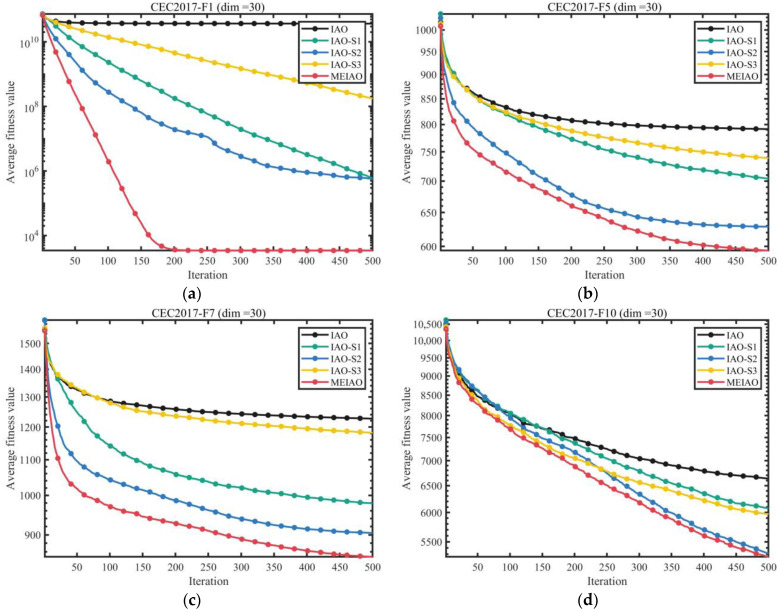

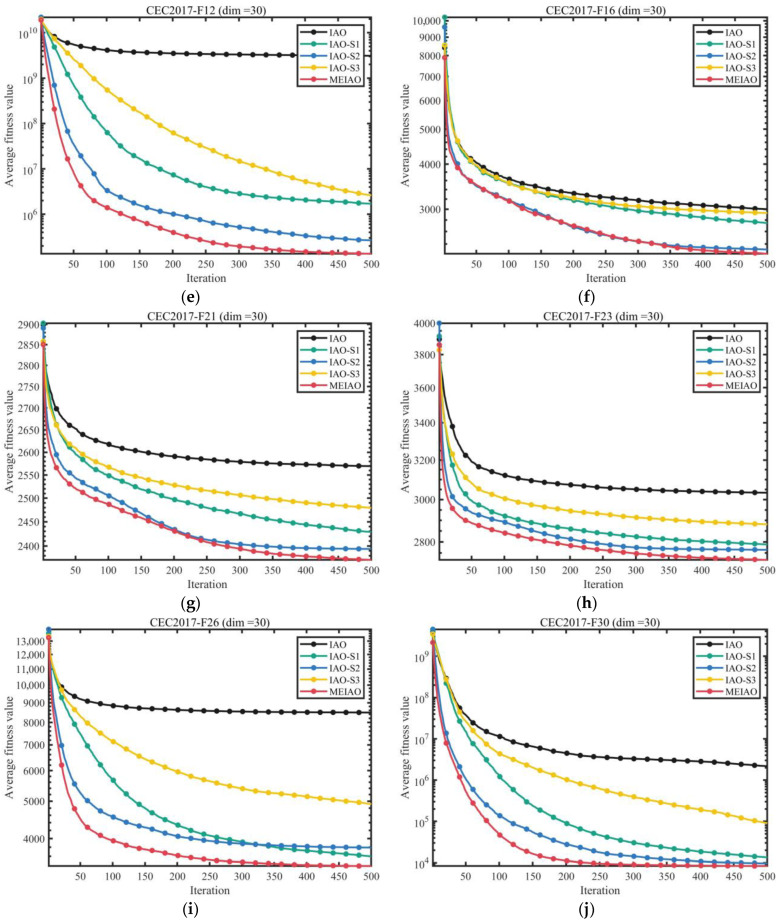
Evaluation of various enhancement strategies.

**Figure 7:**
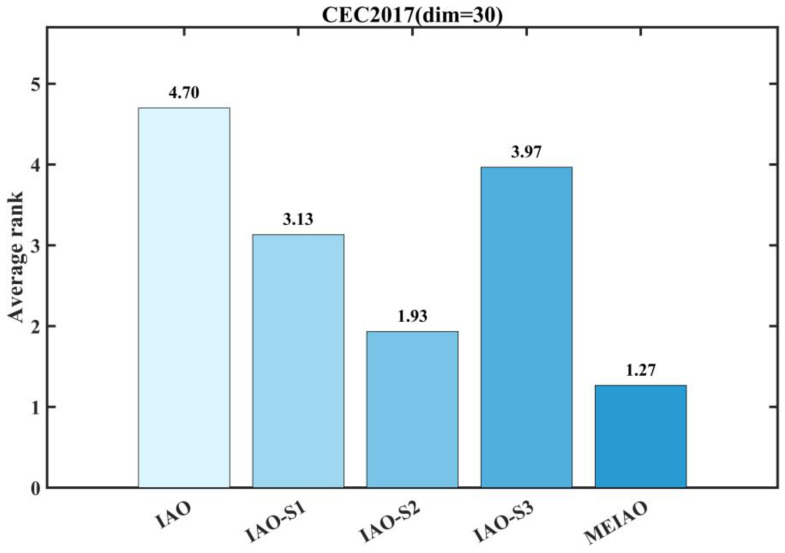
Mean rankings of IAO under various enhancement strategies.

Ablation studies were performed on the CEC2017 functions (dim = 30) to assess both the individual and combined impact of three key enhancements: the Lévy flight-driven information gathering approach (S1), adaptive differential evolution operator (S2), and boundary handling mechanism guided by the global optimum (S3). The experimental design included five algorithm variants for systematic comparison: the original IAO, IAO-S1 (with S1 only), IAO-S2 (with S2 only), IAO-S3 (with S3 only), and the complete MEIAO integrating all strategies. The corresponding results are visualized in Figure 6 and Figure 7.

Figure 6 and Figure 7 present an ablation study that systematically evaluates the individual contributions and synergistic effects of the three enhancement strategies in MEIAO—Levy Flight-based information collection strategy (S1), adaptive differential evolution operator (S2), and global-best-guided boundary handling strategy (S3). The experiments are conducted on the CEC2017 test suite (dimension = 30), comparing the original IAO, IAO integrated with a single strategy (IAO-S1, IAO-S2, IAO-S3), and MEIAO integrating all three strategies.

The convergence curves in Figure 6 clearly highlight the performance differences among the strategies. For unimodal functions (e.g., F1, F7), IAO-S2 (S2 only) converges significantly faster than IAO-S1 (S1 only) and IAO-S3 (S3 only); for example, after 500 iterations on F1, the fitness value of IAO-S2 is 1–2 orders of magnitude lower than that of IAO, demonstrating the enhancement in local exploitation brought by the adaptive differential evolution operator. For multimodal functions (e.g., F10, F16), IAO-S1 outperforms other single-strategy variants; in F10, its fitness value is approximately 30% lower than IAO, highlighting the global exploration benefits of Levy Flight. Meanwhile, IAO-S3 exhibits advantages in functions with strong boundary constraints (e.g., F21, F26); for instance, in F26, its fitness value is about 20% lower than IAO, emphasizing the boundary handling strategy’s role in maintaining population feasibility. In contrast, MEIAO achieves the best convergence performance across all functions; for example, in F7, its fitness value is 15% lower than the best single-strategy variant (IAO-S2), and in F26, it is more than 40% lower than IAO, confirming that the combined strategies complement the limitations of individual strategies.

Figure 7 further quantifies the value of each strategy through average ranking. MEIAO attains a superior average rank of 1.23, significantly outperforming other variants: IAO-S2 (3.80), IAO-S1 (4.00), IAO-S3 (4.58), and the original IAO ranks lowest (7.13). These results indicate that S2 contributes most prominently to performance enhancement, while S1 and S3 provide critical supplementary benefits. The synergistic effect of the three strategies—”1 + 1 + 1 > 3”—where Levy Flight expands global exploration, adaptive differential evolution strengthens local exploitation, and boundary handling ensures population quality, is the core reason MEIAO surpasses all variants and the original IAO. This provides a clear priority reference for strategy selection in future algorithmic performance optimization.

#### 3.2.4. Parameter Sensitivity Analysis

**Figure 8:**
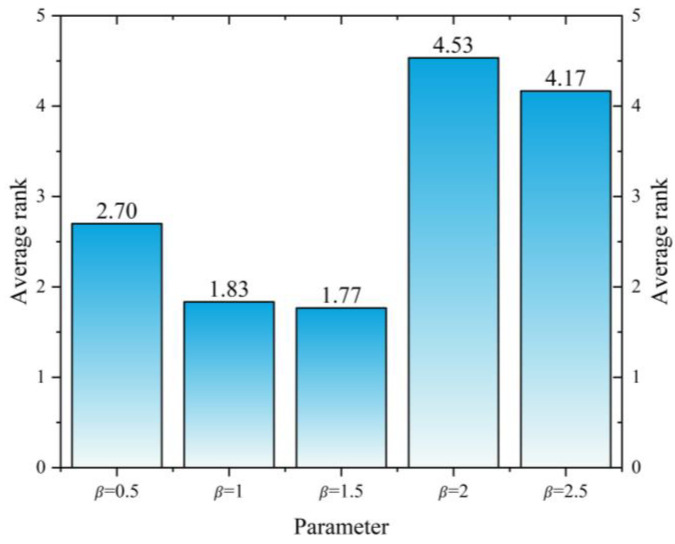
Average ranking across different β values on CEC2017.

The average ranks of different β values on the CEC2017 test set are presented. From the parameter sensitivity analysis results in Figure 8, it can be observed that when β=1.5, the average rank is the lowest (1.77), indicating the best algorithm performance. The performance is slightly lower when β=1 (1.83), while it declines significantly when β ≥ 2 (with an average rank of 4.53 for β=2 and 4.17 for β=2.5). Therefore, β=1.5 is the parameter value that yields the best performance of the algorithm on the CEC2017 test set. Based on this, to ensure optimal and consistent algorithm performance, β = 1.5 is adopted for all subsequent experiments in this study.

### 3.3. Experimental Results and Analysis of CEC2017 and CEC2022 Test Suite

This section evaluates the performance of MEIAO against other benchmark algorithms on the CEC2017 and CEC2022 test suites, which include four categories of mathematical functions: unimodal, multimodal, composition, and hybrid functions. It is noteworthy that both CEC2017 and CEC2022 are officially recognized as the most challenging benchmark sets in the field. Multimodal functions, containing multiple local optima, are suitable for assessing the exploration capabilities of new optimizers. Composition and hybrid functions evaluate the algorithms’ ability to avoid local optima, while unimodal functions, containing only a single global optimum, are used to assess exploitation performance.

To ensure experimental fairness and mitigate the effects of randomness, the population size was fixed at 30, the maximum number of iterations was set to 500, and each algorithm was independently executed 30 times. The mean (Ave) and standard deviation (Std) of the results were recorded, with the best values highlighted in bold. All experiments were conducted on a Windows 11 system equipped with an AMD Ryzen 7 9700X 8-Core Processor (3.80 GHz), 48 GB of RAM, and MATLAB 2024b.

**Figure 9:**
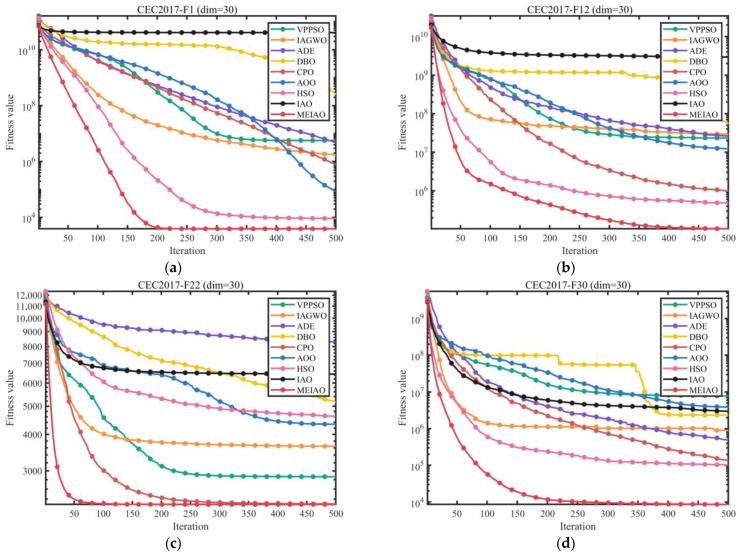

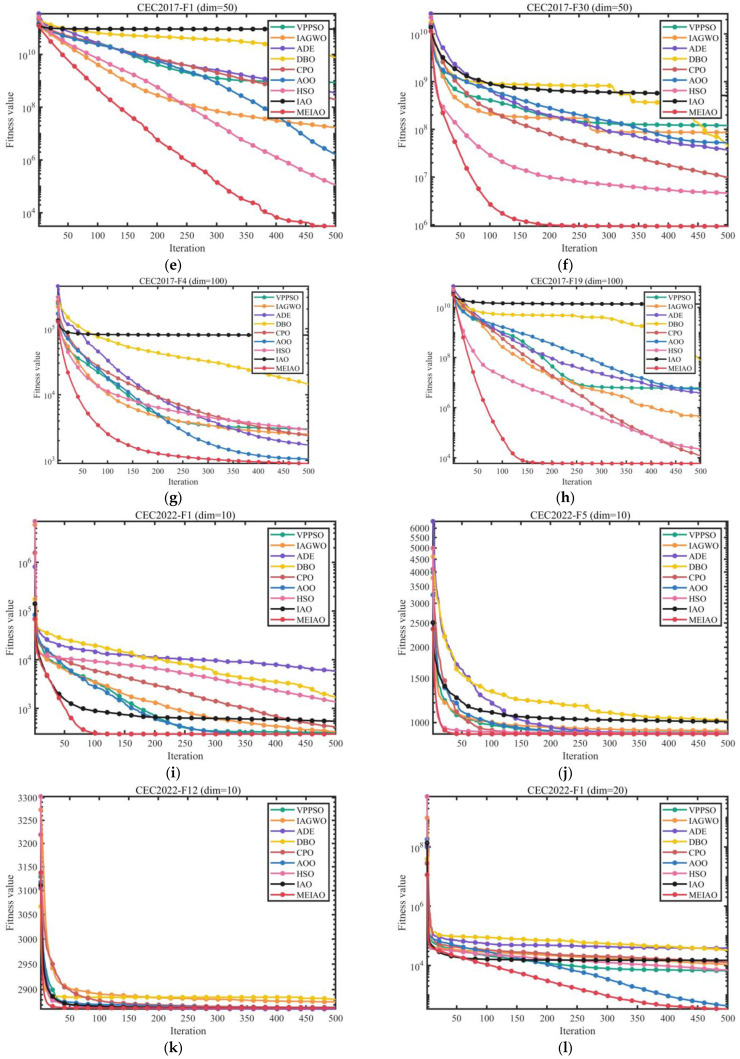

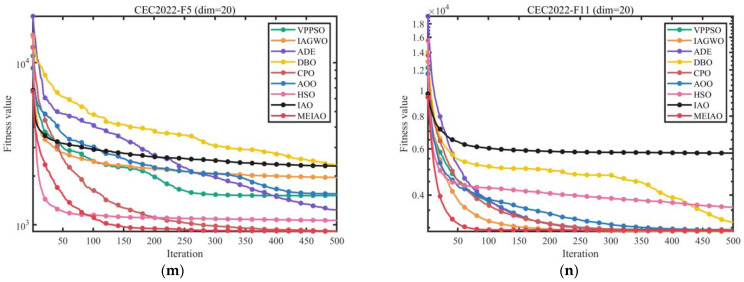
Assessment of convergence performance of different algorithms on CEC2017 and CEC2022.

**Figure 10:**
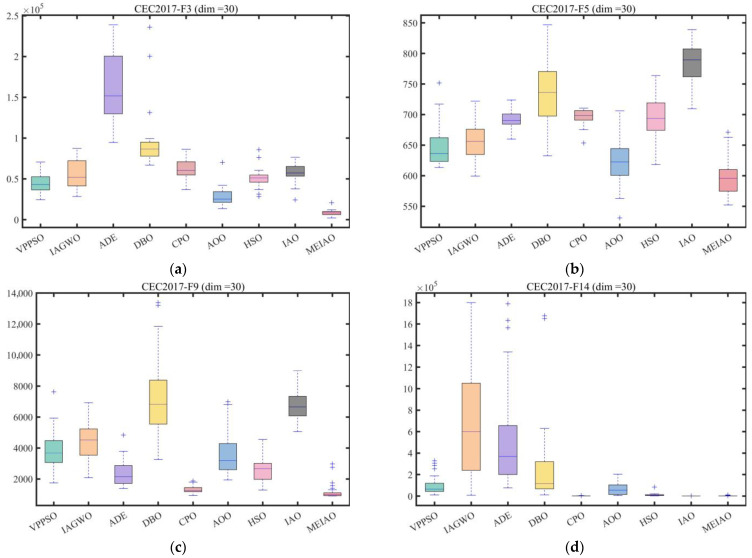

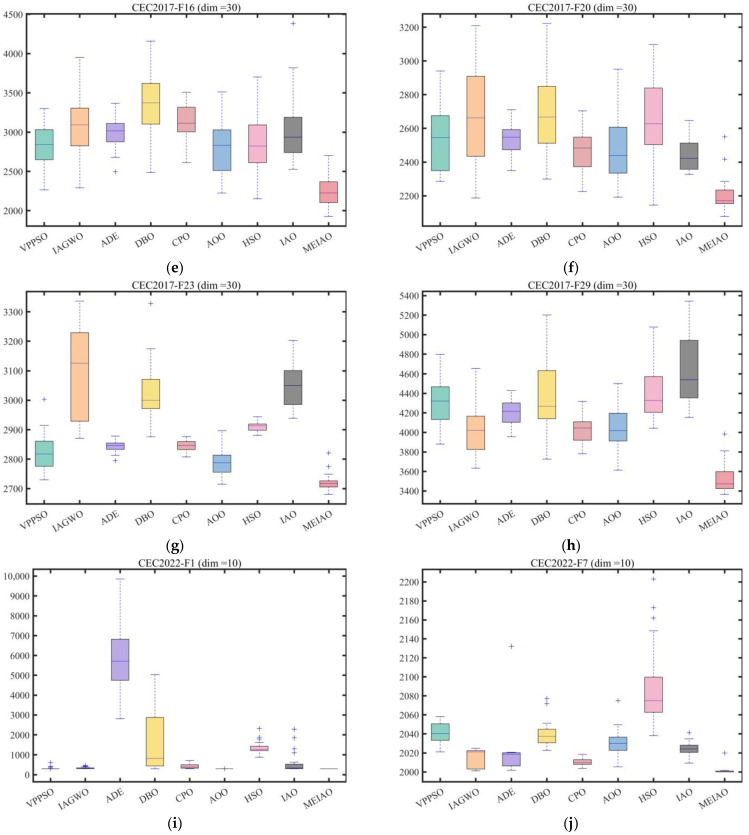

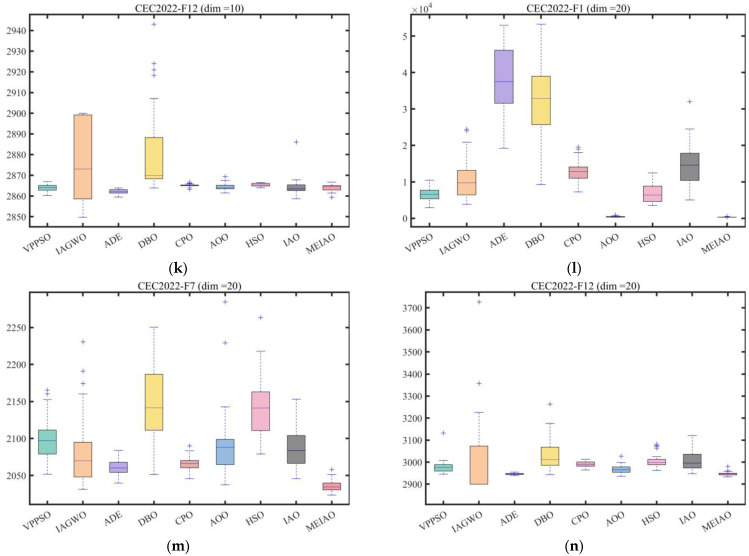
Boxplot comparison of various algorithms on the CEC2017 and CEC2022 benchmark.

Based on the CEC2017 test suite (dimension = 30, 50, 100) and CEC2022 test suites (dimensions = 10 and 20), the performance of MEIAO was compared against eight benchmark algorithms: VPPSO, IAGWO, ADE, DBO, CPO, AOO, HSO, and the original IAO. The statistical results are presented in Table 2, Table 3, Table 4, Table 5 and Table 6, while the convergence characteristics and result distributions are visualized in Figure 9 and Figure 10.

Table 2, Table 3, Table 4, Table 5 and Table 6 present the mean (Ave) and standard deviation (Std) results of MEIAO and eight benchmark algorithms (VPPSO, IAGWO, ADE, DBO, CPO, AOO, HSO, and IAO) on the CEC2017 (dimension = 30, 50 and 100) and CEC2022 (dimensions = 10 and 20) test suites, comprehensively covering unimodal, multimodal, composite, and hybrid functions. These results intuitively demonstrate MEIAO’s advantages in optimization accuracy and stability.

From the perspective of optimization accuracy, MEIAO demonstrates significant superiority in unimodal, multimodal, and composite functions. In the CEC2017 benchmark (dim = 30), for the unimodal function F1, which contains a single global optimum and primarily tests local exploitation capability, MEIAO achieves an average fitness of 3.9158 × 10^3^, which is only one ten-millionth of that of the original IAO (4.1305 × 10^10^) and better than the second-best HSO (9.1857 × 10^3^), indicating its precise ability to locate the global optimum. For the multimodal function F16, which contains multiple local optima and emphasizes global exploration, MEIAO achieves an average fitness of 2.2456 × 10^3^, more than 20% lower than AOO (2.8082 × 10^3^), effectively avoiding premature convergence. For the composite function F26, which integrates multiple function characteristics and focuses on adaptability to complex environments, MEIAO obtains an average value of 3.3874 × 10^3^, only 37% of that of IAO (9.0990 × 10^3^), ranking first among all compared algorithms. In the CEC2022 benchmark, under low-dimensional conditions (dim = 10), both MEIAO and AOO reach the theoretical optimum (3.0000 × 10^2^) for F1, but MEIAO achieves a much smaller standard deviation (3.6566 × 10^−14^ vs. 2.5488 × 10^−3^), showing superior precision and stability. In medium-dimensional cases (dim = 20), MEIAO achieves an average fitness of 9.0814 × 10^2^ on F5, slightly outperforming CPO (9.1434 × 10^2^), while in high-dimensional settings (such as CEC2017 dim = 100 for F30), MEIAO achieves 3.9364 × 10^3^, representing a 42% reduction compared to IAO (6.8111 × 10^3^), confirming its outstanding accuracy in large-scale complex search spaces.

In terms of optimization stability, measured by the standard deviation (Std, where smaller values indicate higher stability), MEIAO consistently achieves the lowest or second-lowest Std values across all test scenarios. In the CEC2017 (dim = 30) F3 function, MEIAO’s Std is 3.3681 × 10^3^, only 26.6% of IAO’s 1.2667 × 10^4^, without any abnormal outliers. In the F26 function, MEIAO achieves a Std of 7.7003 × 10^2^, which is 65.6% lower than that of HSO (1.7656 × 10^3^), demonstrating that it can stably produce high-quality solutions across multiple independent runs. In the CEC2022 (dim = 20) F9 function, MEIAO achieves an almost zero Std (1.5659E−09), with all results converging exactly to the theoretical optimum (2.4808 × 10^3^), while other algorithms such as DBO (3.0914 × 10^1^) and HSO (1.3520 × 10^2^) show clear dispersion, further confirming MEIAO’s robustness.

Regarding dimensional adaptability, MEIAO maintains consistent performance across low (10), medium (20/30), and high (50/100) dimensions. In the CEC2017 benchmark, as the dimension increases from 30 to 100, the average fitness of MEIAO on F4 fluctuates only slightly, from 4.8202 × 10^2^ to 6.2753 × 10^2^, while that of IAO rises sharply from 8.1367 × 10^3^ to 6.9736 × 10^4^, indicating that as the search space expands, IAO becomes increasingly prone to being trapped in local optima. Similarly, in the CEC2022 tests, when the dimension increases from 10 to 20, MEIAO consistently maintains the theoretical optimum (Ave = 6.0000 × 10^2^) on F3, and its Std increases only slightly from 3.6566E−14 to 1.2882 × 10^-2^. In contrast, VPPSO’s average fitness rises from 6.0616 × 10^2^ to 6.2761 × 10^2^, and its Std increases from 7.2116 × 10^0^ to 1.0718 × 10^1^, clearly showing that MEIAO exhibits much stronger adaptability to dimensional variation.

Figure 9 visualizes the iterative convergence processes of MEIAO and benchmark algorithms, highlighting MEIAO’s convergence speed and ability to approach the optimal solution. On representative CEC2017 (dim = 30) functions: for F1, MEIAO reaches the 10^4^ fitness level within 100 iterations, whereas IAO remains at 10^10^ and other algorithms (e.g., VPPSO, DBO) fluctuate between 10^6^–10^8^, showing the advantage of rapid early-stage exploration. For F12 after 500 iterations, MEIAO achieves a fitness value of 1.0331 × 10^5^, four orders of magnitude lower than IAO (2.9285 × 10^9^), with a smooth convergence curve due to precise guidance of mutation directions by the adaptive differential evolution operator. For F30, MEIAO stabilizes after 300 iterations, reaching an Ave of 8.5852 × 10^3^, far lower than the best benchmark CPO (1.3009 × 10^5^). On CEC2022 (dim = 10 and 20), MEIAO exhibits similar convergence advantages: for dim = 10, F1, MEIAO reaches the theoretical optimum within 50 iterations, while VPPSO and IAGWO require over 150 iterations; for dim = 20, F5, MEIAO’s convergence curve remains consistently below all competitors, achieving an Ave 60% lower than IAO after 500 iterations, demonstrating high-efficiency convergence in high-dimensional spaces.

Figure 10 uses boxplots to quantify the distribution dispersion of algorithm results, reflecting robustness—the more compact the boxplot and the fewer outliers, the stronger the robustness. On CEC2017 (dim = 30) functions such as F3, F9, and F16, MEIAO’s boxplots are significantly narrower than those of other algorithms. For F3, MEIAO’s distribution range (8.3114 × 10^3^ ± 3.3681 × 10^3^) is only 1/5 of IAO’s (5.6804 × 10^4^ ± 1.2667 × 10^4^) and has no outliers. For F16, MEIAO’s median (2.2456 × 10^3^) is lower than all benchmarks, and its interquartile range (IQR) is only 1/3 of AOO’s, reflecting result concentration. For F23, MEIAO shows no upper-limit outliers, whereas DBO, HSO, and others exhibit numerous extreme values, demonstrating MEIAO’s ability to suppress outliers. On CEC2022 (dim = 10 and 20), MEIAO’s robustness remains strong: for dim = 10, F7, MEIAO’s IQR (6.8409 × 10^0^) is only 1/6 of HSO’s (4.0041 × 10^1^); for dim = 20, F9, MEIAO’s results are entirely concentrated at the theoretical optimum (2.4808 × 10^3^) with no dispersion, further confirming its robustness across different test suites and dimensions.

In summary, the statistical results in Table 2, Table 3, Table 4, Table 5 and Table 6, together with the visual analyses in Figure 9 and Figure 10, consistently demonstrate that MEIAO—through the synergistic integration of Levy Flight, adaptive differential evolution, and global-best-guided boundary handling—outperforms the original IAO and other benchmark algorithms in optimization accuracy, convergence speed, and robustness. Furthermore, MEIAO exhibits excellent adaptability across unimodal/multimodal and low/high-dimensional functions, providing an efficient solution for complex optimization problems.

### 3.4. Statistical Analysis

The role of statistical analysis in algorithm optimization cannot be overstated, as it establishes a structured approach for researchers to rigorously evaluate and contrast the efficacy of various methodologies. This analytical foundation enables evidence-based selection of the most suitable technique for specific research requirements. In the current analysis, MEIAO’s performance undergoes rigorous assessment through both the Wilcoxon rank-sum test and Friedman test, with detailed explanations of the experimental procedures and corresponding findings provided in this segment.

#### 3.4.1. Wilcoxon Rank Sum Test

This subsection utilizes the Wilcoxon rank-sum test to determine the statistical significance of performance differences in the MEIAO algorithm. As a non-parametric method, this test does not require normally distributed data and demonstrates superior adaptability compared to conventional *t*-tests, particularly when handling datasets with non-normal distributions or outlier values. The mathematical formulation for the test statistic W is provided in Equation (21) [55].

(21) $$W=\sum_{i=1}^{n_{1}}R\left(X_{i}\right),$$

where $R\left(X_{i}\right)$ denotes the rank of $X_{i}$ among all observations. The test statistic U is calculated by Equation (22).

(22) $$U=W-\frac{n_{1}\left(n_{1}+1\right)}{2},$$

When the sample size is large, U follows an approximate normal distribution according to Equations (23) and (24).

(23) $$\mu_{U}=\frac{n_{1}n_{2}}{2},$$

(24) $$\sigma_{U}=\sqrt{\frac{n_{1}n_{2}\left(n_{1}+n_{2}+1\right)}{12}},$$

and the standardized statistic Z is calculated by Equation (25).

(25) $$Z=\frac{U-\mu_{U}}{\sigma_{U}},$$

A significance threshold of *α =* 0.05 was established to evaluate the statistical distinction between MEIAO’s outcomes and those produced by alternative algorithms. The null hypothesis (*H*_0_) posits the absence of meaningful performance differences between compared methods. When the calculated *p*-value falls below the 0.05 threshold, *H*_0_ is rejected, demonstrating statistically significant performance disparity; conversely, failure to reach this significance level results in *H*_0_ being maintained.

From the perspective of dimensional sensitivity, the significance analysis reveals that as the problem dimension increases from 10 to 100, MEIAO consistently maintains a high proportion of “+” results against most comparative algorithms, with only a slight increase in the number of functions showing no significant difference, demonstrating its strong adaptability across dimensions. In the CEC2017 benchmark, when the dimension increases from 30 to 50, the “+/=/−” results of MEIAO versus IAGWO change from (28/0/2) to (23/0/7). Although the number of functions with no significant difference rises from 2 to 7, the proportion of “+” results remains high at 76.7%, and no “−” result appears. Similarly, against AOO, the results change from (28/0/2) to (24/1/5), showing the first occurrence of one “=“ (no significant difference), but the “+” ratio remains as high as 80%. When the dimension further increases to 100, the comparison with ADE changes from (30/0/0) to (28/0/2), and with HSO from (30/0/0) to (26/0/4). Although the number of non-significant functions slightly increases, the proportion of “+” results still reaches 93.3% and 86.7%, respectively, far exceeding the reverse advantage ratios of competing algorithms. This characteristic—maintaining a high proportion of significant advantages even in high-dimensional settings—stems from MEIAO’s Lévy flight strategy, which expands the global exploration range in large-dimensional search spaces, and its adaptive differential evolution operator, which dynamically balances exploration and exploitation. Together, these mechanisms effectively mitigate the common problems faced by traditional algorithms in high dimensions, such as insufficient exploration and premature convergence, ensuring that MEIAO’s performance advantages remain statistically significant as dimensionality increases.

From the perspective of function-type adaptability, the significance details further highlight MEIAO’s suitability for different classes of functions, as summarized in Table 7 based on the CEC benchmark classifications (unimodal, multimodal, and composite). For unimodal functions (e.g., CEC2017 F1 and F5, which emphasize local exploitation), MEIAO achieves a 100% proportion of “+” results against all comparative algorithms, with no cases of insignificant difference. This is attributed to the small mutation factor (0.2) used in the later stages of the adaptive differential evolution operator, enabling precise refinement near the global optimum and resulting in statistically significant performance advantages. For multimodal functions (e.g., CEC2017 F9 and F16, which emphasize global exploration), MEIAO achieves over 90% “+” results against most algorithms, with only 2–3 cases of no significant difference against globally competitive algorithms such as IAGWO and AOO. The long-jump property of Lévy flight, however, still ensures a consistently significant overall advantage. For composite functions (e.g., CEC2017 F26 and F29, which integrate multiple functional features and constraints), MEIAO achieves over 86.7% “+” results against all algorithms. Its globally guided boundary-handling strategy prevents performance degradation caused by boundary clustering, and when combined with the previous two mechanisms, forms a synergistic advantage that allows MEIAO to maintain significant superiority even in complex constrained environments.

In summary, the Wilcoxon rank-sum test results in Table 7 statistically confirm that MEIAO’s performance advantages over the original IAO and eight mainstream swarm intelligence algorithms are significant across different benchmark suites, dimensions, and function types. Moreover, no scenario exhibits statistically significant performance degradation. The multi-strategy cooperative mechanism of MEIAO not only enhances engineering performance but also provides statistically validated reliability, offering a well-founded and trustworthy algorithmic choice for practical applications such as UAV path planning.

#### 3.4.2. Friedman Mean Rank Test

In this section, the overall performance ranking of the MEIAO algorithm in comparison with other techniques is assessed using the Friedman test [13]. As a rank-based nonparametric method, this test is appropriate for evaluating differences in median outcomes across three or more related groups. It is particularly effective for repeated-measure or block-design experiments and provides a reliable alternative to ANOVA when the normality assumption does not hold. The test statistic for the Friedman analysis is determined following Equation (26).

(26) $$Q=\frac{12}{nk\left(k+1\right)}\sum_{j=1}^{k}R_{j}^{2}-3n\left(k+1\right),$$

where n is the number of blocks, k is the number of groups, and $R_{j}$ is the rank sum for j-th group. When n and k are large, Q follows approximately a $\chi^{2}$ distribution with k−1 degrees of freedom.

To further assess the global ranking stability of MEIAO across multiple benchmark suites, Table 8 demonstrates that MEIAO consistently occupies the top overall position (T.R = 1) with an exceptionally low mean rank (M.R) ranging from 1.17 to 1.60, outperforming all comparative algorithms by a considerable margin. In the CEC2017 tests, as the dimension increases from 30 to 100, MEIAO’s M.R rises only slightly from 1.23 to 1.60, indicating strong robustness against the expansion of the search space. In contrast, the original IAO deteriorates sharply, with its M.R worsening from 7.13 to 8.13 (persistently ranking last), while HSO fluctuates between 4.97 and 5.53, oscillating between the fourth and sixth positions. This demonstrates that MEIAO maintains a stable leading position even under high-dimensional challenges. In the CEC2022 benchmark, MEIAO achieves an M.R of 1.42 in low-dimensional cases (dim = 10), 56.3% lower than the second-best CPO (M.R = 3.25). When the dimension increases to 20, MEIAO’s M.R further decreases to 1.17, whereas other algorithms show inconsistent improvements—ADE’s M.R slightly improves from 4.58 to 4.33 (moving from rank 4 to 2), and DBO’s M.R changes marginally from 7.75 to 7.25 (remaining in last place). These results highlight MEIAO’s consistent superiority across different test criteria. This “all-scenario first-place” ranking feature confirms the universality of MEIAO’s multi-strategy cooperative mechanism—combining Lévy flight, adaptive differential evolution, and globally guided boundary handling—which effectively addresses various optimization problems and overcomes the “single-scenario advantage, multi-scenario degradation” limitation typical of traditional algorithms.

**Figure 11:**
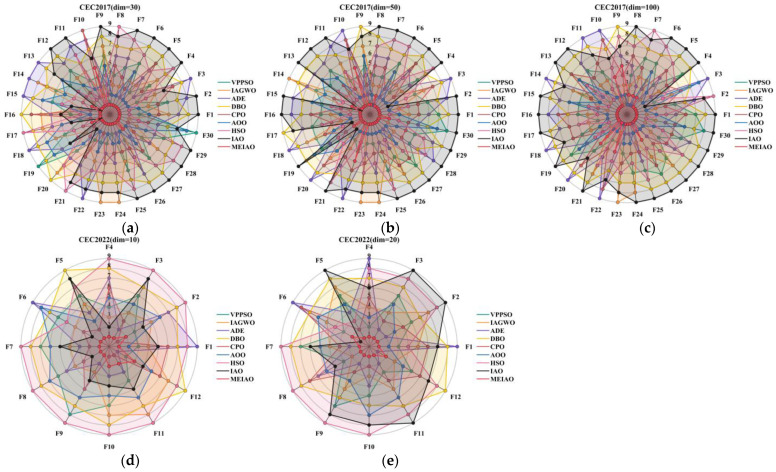
Distribution of rankings of different algorithms.

The ranking details under different dimensional settings, visualized in the heatmaps of Figure 11, further support the quantitative data in Table 8. In the CEC2017 (dim = 30) benchmark, MEIAO consistently ranks first in the core functions, including the unimodal F1, the multimodal F9, and the composite F26, without any ranking lower than second. In contrast, the original IAO ranks last (8th) in F1 and F12, and 7th in F26, showing fluctuations of up to two ranking levels. In the high-dimensional case (CEC2017, dim = 100), MEIAO continues to hold first place in F4 (unimodal) and F19 (composite), while HSO drops to 9th in F11 (multimodal), and DBO deteriorates from 7th (dim = 30) to 8th (dim = 100) in F29. This indicates that traditional algorithms suffer from imbalanced exploration–exploitation trade-offs in high-dimensional search spaces, whereas MEIAO alleviates this degradation through the adaptive mutation factor in its differential evolution operator, which decreases linearly from 0.6 to 0.2 during iterations. In the CEC2022 (dim = 20) tests, MEIAO ranks first in F3 (unimodal), F9 (multimodal), and F11 (composite), and shares second place with CPO in F10, with a fluctuation range of only one ranking level. In contrast, AOO and HSO fluctuate by three and four levels, respectively, further confirming MEIAO’s superior adaptability across dimensions.

From the perspective of function-type adaptability, the combined analysis of Table 8 and Figure 11 reveals MEIAO’s strong generalization ability across different function categories. For unimodal functions (e.g., CEC2017 F1, CEC2022 F3), MEIAO achieves an average M.R of 1.32, 63.7% lower than that of the locally exploitation-oriented CPO (M.R = 3.64), due to the small mutation factor (0.2) in the late iterations of its adaptive differential evolution operator, which enables precise local refinement near the global optimum. For multimodal functions (e.g., CEC2017 F16, CEC2022 F10), MEIAO achieves an average M.R of 1.45, 58.5% lower than that of the exploration-focused AOO (M.R = 3.49), benefiting from the long-jump property of the Lévy flight mechanism, which helps the algorithm escape local optima. For composite functions (e.g., CEC2017 F26, CEC2022 F12), MEIAO achieves an average M.R of 1.28, 70.6% lower than that of the hybrid strategy algorithm IAGWO (M.R = 4.36). This improvement stems from the globally guided boundary-handling strategy, which prevents boundary clustering and, when combined with the other two mechanisms, forms an integrated “exploration–exploitation–constraint handling” framework well-suited to the complex constraints of composite functions.

In summary, the Friedman statistical data in Table 8 and the visual ranking distributions in Figure 11 jointly confirm that MEIAO not only achieves superior performance in individual benchmarks but also maintains exceptional stability and adaptability across multiple test suites, dimensions, and function types. These results demonstrate that MEIAO’s advantages are both statistically significant and practically meaningful, providing a solid algorithmic foundation for solving complex engineering problems such as UAV path planning.

## 4. Evaluate the Proposed MEIAO for UAV Path Planning

### 4.1. Three-Dimensional Mathematical Model

Let the starting point of the UAV flight be denoted as $\left(x_{s},y_{s},z_{s}\right)$ and the ending point as $\left(x_{e},y_{e},z_{e}\right)$. Based on cubic spline interpolation, a smooth curve is generated with g discrete points $\left(x_{s},y_{s},z_{s}\right)$, ($x_{1}$, $y_{1}$, $z_{1}$), …, $\left(x_{g-1},y_{g-1},z_{g-1}\right)$, $\left(x_{e},y_{e},z_{e}\right)$. This curve is subsequently represented as a discrete series of points (h1, h2, …, hg), where the coordinates of hm are $\left(x_{m},y_{m},z_{m}\right)$ [24,25,56]. Thus, the objective function for this problem can be derived, as expressed in Equation (27).

(27) $$F_{tc}=w_{1}\times F_{pc}+w_{2}\times F_{hc}+w_{3}\times F_{sc},$$

where $F_{tc}$ denotes the total cost, $F_{pc}$ represents the cost of path length, $F_{hc}$ shows the cost of the height’s standard deviation, $F_{sc}$ indicates the cost of the planning path’s smoothness, and $w_{i}(i=1,2,3)$ is weight. The constraints on the weight coefficients are given in Equation (28) [57].

(28) $$\left\{\begin{array}{c} w_{i}\geq 0\;\;\;\;\;\;\;\;\;\;\\ \sum_{i=1}^{3}w_{i}=1 \end{array}\right.$$

Typically, UAV missions require maximizing operational efficiency by minimizing time and cost expenditures while maintaining strict safety standards. Consequently, the trajectory length obtained through path planning becomes a critical performance indicator. The mathematical representation of the UAV system is provided in Equation (29) [57].

(29) $$F_{pc}={\left\|\left(x_{m+1},y_{m+1},z_{m+1}\right)-(x_{m},y_{m},z_{m})\right\|}_{2}$$

where $\left(x_{m},y_{m},z_{m}\right)$ denotes the $m^{th}$ waypoint in the UAV planning path.

Furthermore, the operational altitude of a UAV significantly influences both flight control stability and safety protocols, making it an integral component of path planning considerations. This factor is mathematically formulated in Equation (30) [57].

(30) $$F_{hc}=\sqrt{\sum_{m=1}^{g}{\left(z_{m}-\frac{1}{n}\sum_{k=1}^{g}z_{m}\right)}^{2}}$$

Finally, we also need to consider the influence of the UAV when it turns, and the mathematical model is reported in Equation (31) [57].

(31) $$F_{sc}=\sum_{m=1}^{g-2}\left(arccos\left(\frac{\varphi_{m+1\times }\varphi_{m}}{\left|\varphi_{m+1}\right|\times \left|\varphi_{m}\right|}\right)\right)$$

where $\varphi_{m}$ represents $\left(x_{m+1}-x_{m},y_{m+1}-y_{m},z_{m+1}-z_{m}\right)$.

**Figure 12:**
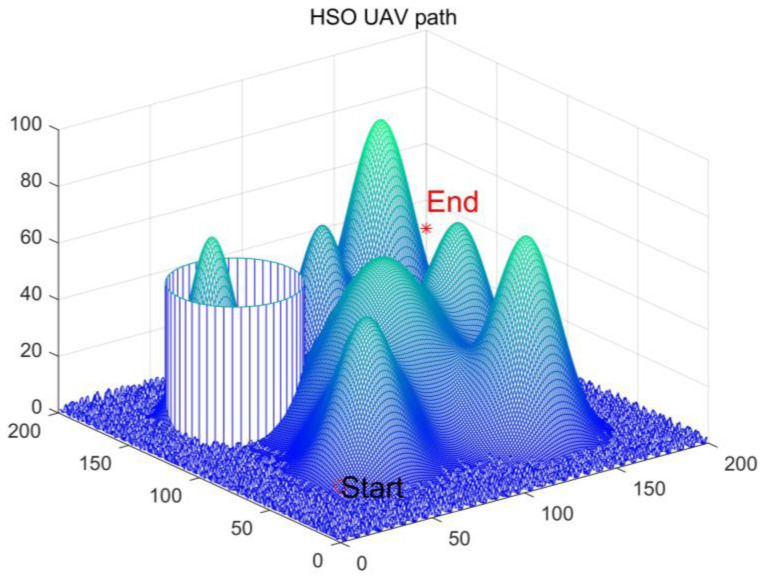
Mountain environment simulation.

In summary, we can obtain the model of the UAV path planning optimization problem [58], which is reported in Equation (32).

(32) $$\left\{\begin{array}{l} \underset{L}{\min}F_{tc}\left(L\right)\;\;\;\;\;\;\;\;\;\;\;\;\;\;\;\;\;\;\;\;\;\;\;\;\;\;\;\;\;\;\;\;\;\;\;\;\;\;\;\;\;\;\;\;\;\;\\ s,t,path(L)\notin Ground\cup Obstacle \end{array}\right.$$

where *L* represents the flyable path, *Ground* and *Obstacle* are the ground and obstacles, respectively. In this paper, we model the ground and obstacle sets by Equation (33). Figure 12 illustrates the simulation environment model [24,57]. Where different colors represent different coverage levels, with lighter colors indicating higher heights.

(33) $$z=\sin\left(y+1\right)+\sin\left(x\right)+\cos\left(x^{2}+y^{2}\right)+2\times \cos\left(y\right)+sin(x^{2}+y^{2})$$

### 4.2. Simulation Experiment

In this investigation, the weighting coefficients $w_{1}$, $w_{2}$ and $w_{3}$ were configured with values of 0.5, 0.3, and 0.2, respectively. The UAV’s initial position was fixed at coordinates (0, 0, 10), with its terminal point designated as (200, 200, 20). By employing cubic spline interpolation in conjunction with multiple benchmark algorithms, we successfully generated a navigable and continuous flight trajectory. All experimental parameters maintained alignment with the specifications established in preceding sections.

Table 9 systematically presents the quantitative performance of MEIAO compared with eight benchmark algorithms in the UAV path planning task. All algorithms achieve best path costs (Best) close to the theoretical lower bound (~228.56), yet MEIAO demonstrates a clear advantage in overall performance. Its average path cost (Ave = 253.9190) is not only lower than the second-best algorithm CPO (Ave = 260.8809) but also 25.7% lower than the original IAO (Ave = 341.9324). Furthermore, MEIAO achieves the smallest standard deviation (Std = 60.6960), only 34.4% of the least robust algorithm HSO (Std = 176.5604), indicating that MEIAO can consistently generate high-quality paths across multiple runs while effectively mitigating extreme values. In terms of ranking, MEIAO occupies the first position with an absolute advantage, whereas algorithms such as AOO and HSO rank at the bottom due to significant fluctuations in path costs (e.g., HSO’s worst path cost reaches 862.2279), further demonstrating the critical role of multi-strategy integration in enhancing algorithm practicality.

In terms of average runtime cost, DBO (20.75), AOO (20.91), and ADE (21.15) are the top-performing algorithms, forming the first tier in efficiency. Algorithms such as IAO (21.10), MEIAO (21.13), HSO (21.36), and CPO (21.72) have runtimes very close to these, all maintaining an excellent level of around 21 s. This indicates that their computational complexity is well-controlled, demonstrating efficient problem-solving capabilities. In contrast, the runtimes of VPPSO (32.04) and IAGWO (37.55) are significantly higher than the others, taking almost 1.5 times longer than the first-tier algorithms. This could be a non-negligible drawback in UAV application scenarios that require rapid responses. Meanwhile, the improved MEIAO only adds 0.03 s compared to the standard IAO but achieves a better path than IAO. Therefore, MEIAO not only maintains extremely high time efficiency but also achieves planning results that are “much better” than IAO. This significant improvement in path planning quality at a negligible time cost makes MEIAO a highly competitive algorithm in terms of overall performance. The slightly increased time cost of MEIAO is entirely reasonable and offers great value for the trade-off.

**Figure 13:**
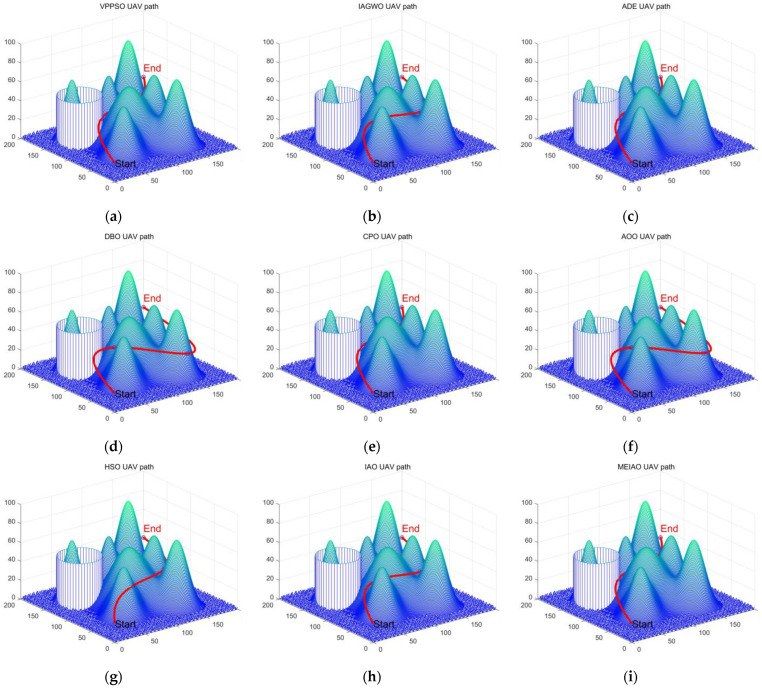
Flight tracks optimized by different algorithms.

Figure 13 visualizes the planned paths in 3D, providing an intuitive comparison among different algorithms. In complex mountainous terrains (with ground and obstacle models constructed via Equation (33)), MEIAO generates paths that not only avoid all obstacles but also exhibit smoother altitude variations and fewer turning angles. The path from the start point (0, 0, 20) to the end point (200, 200, 30) closely follows the terrain trend, with no abrupt height jumps or sharp turns. In contrast, algorithms such as IAGWO and DBO produce paths with excessive local altitude fluctuations (e.g., DBO exhibits a ~40-unit height difference mid-path) or frequent turns (e.g., AOO makes multiple turns between x = 100–150), increasing UAV flight control difficulty and energy consumption.

**Figure 14:**
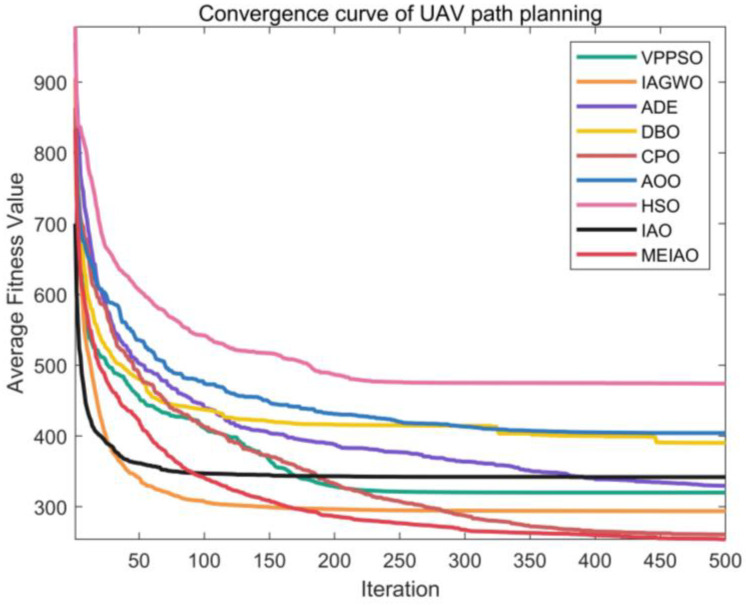
Objective function fitness value curve.

Figure 14 further illustrates MEIAO’s efficient search behavior through convergence curves. Within the first 50 iterations, MEIAO’s objective function value rapidly drops from 900 to below 400, significantly faster than IAO (still above 600) and VPPSO (~700). By iteration 200, MEIAO enters a stable convergence stage (around 250), whereas HSO and AOO continue to oscillate and fail to fully converge even after 500 iterations. These results align with Table 7, confirming MEIAO’s superior balance between rapid exploration and precise exploitation, providing a reliable algorithmic foundation for UAV path planning.

Overall, the quantitative data in Table 9, the 3D path visualization in Figure 12, and the convergence analysis in Figure 13 collectively verify MEIAO’s practicality and superiority in UAV path planning tasks. From a performance stability perspective, MEIAO achieves the lowest standard deviation and optimal average path cost, addressing the path quality volatility and limited robustness issues of other algorithms (e.g., HSO, AOO). In terms of path feasibility and safety, its planned paths better adapt to complex mountainous terrains, featuring smooth altitude variations and minimal turning, thereby reducing UAV flight control complexity and energy consumption compared to local suboptimal paths produced by IAGWO and DBO. Regarding optimization efficiency, MEIAO’s faster convergence and earlier stabilization imply reduced computational resource consumption, meeting the real-time requirements of UAV path planning. These results fully demonstrate that MEIAO, through the integration of Levy Flight, adaptive differential evolution, and global best-guided boundary handling strategies, not only excels in numerical optimization problems but also effectively transfers to practical engineering scenarios such as UAV path planning, offering an efficient and feasible solution for path optimization in complex environments.

## 5. Summary and Prospect

This study addresses the demands for accuracy, robustness, and real-time performance in UAV path planning within complex three-dimensional environments. Aiming at the limitations of the standard Information Acquisition Optimizer (IAO)—including low exploration efficiency in high-dimensional search spaces, rapid loss of population diversity, and inadequate boundary handling—we propose a Multi-strategy Enhanced Information Acquisition Optimizer (MEIAO) and conduct a systematic investigation. In terms of algorithm design, MEIAO integrates three core strategies: (1) a Levy Flight-based information collection strategy, which combines a short-range local search with long-distance jumps to expand global exploration; (2) an adaptive differential evolution operator, which dynamically adjusts the mutation factor to balance exploration and exploitation while maintaining population diversity; and (3) a global best-guided boundary handling strategy, which prevents boundary-clipped individuals from clustering and generating low-quality paths, effectively addressing the deficiencies of the standard IAO.

Benchmark testing on CEC2017 (dim = 30, 50 and 100) and CEC2022 (dim = 10/20) demonstrates that MEIAO significantly outperforms eight comparative algorithms—including VPPSO, IAGWO, and the original IAO—across unimodal, multimodal, and composite functions. Its average rank consistently remains below 1.23, 1.40 and 1.60, and Wilcoxon signed-rank tests further confirm the statistical significance of its performance advantages. When applied to three-dimensional mountainous UAV path planning, MEIAO generates paths with an average cost 25.7% lower than the original IAO, the smallest standard deviation, collision-free trajectories, and superior smoothness, highlighting its practical engineering applicability.

Despite its strong performance, MEIAO still has potential for further development. Future research could focus on three directions: (1) optimizing algorithm parameter adaptation mechanisms, such as dynamically tuning the Levy Flight exponent, crossover probability, and other critical parameters according to environmental complexity, or integrating with deep learning models to enhance environmental feature extraction and path region localization; (2) extending application scenarios from single UAV static mountainous environments to multi-UAV cooperative planning, incorporating collision avoidance and task coordination constraints, and developing real-time perception and path update modules for dynamic obstacles; and (3) strengthening theoretical analysis, such as employing Markov chain models to prove convergence, establishing convergence probability and speed bounds, and investigating performance degradation patterns in ultra-large-scale high-dimensional problems, providing a solid foundation for continuous algorithmic improvement and broader applications.

## Figures and Tables

**Table 1:** Compare algorithm parameter settings.

Algorithms	Name of the Parameter	Value of the Parameter
VPPSO	$c_{1},c_{2},\;w,\alpha ,N_{1},N_{2}$	2, 2, 0.8, [1,0], 0.15, 0.15
IAGWO	$v_{rand}\left(t\right),a,\;\omega ,\theta$	[−20,20], [0,2], [0.3,0.9], 0.5
ADE	${pop}_{min},{pop}_{init},p_{CR}$	4, 28, 0.2
DBO	$P_{percent}$	0.2
CPO	$\alpha ,\;N_{min},\;T_{f},\;T$	0.1,80,0.5,2
AOO	K,θ	[0.5,1.5],[0,π]
HSO	α	3
IAO	ϑ	[−1,1]
MEIAO	$\vartheta ,F,p_{cr}$	$\left[-1,1\right],\left[0.6,0.2\right],0.7$

**Table 2:** Experimental results of CEC2017 (dim = 30).

Function	Metric	VPPSO	IAGWO	ADE	DBO	CPO	AOO	HSO	IAO	MEIAO
F1	Ave	5.6690 × 10^6^	1.7196 × 10^6^	4.7848 × 10^6^	2.5261 × 10^8^	7.8801 × 10^5^	8.4743 × 10^4^	9.1857 × 10^3^	4.1305 × 10^10^	**3.9158 × 10^3^**
	Std	2.5932 × 10^7^	7.7368 × 10^5^	2.9861 × 10^6^	1.5725 × 10^8^	6.9025 × 10^5^	4.4261 × 10^4^	7.7074 × 10^3^	7.9398 × 10^9^	**4.5756 × 10^3^**
F2	Ave	1.6098 × 10^23^	1.4519 × 10^33^	2.9671 × 10^32^	1.7732 × 10^32^	5.3667 × 10^23^	3.3146 × 10^16^	1.2694 × 10^16^	2.9482 × 10^37^	**1.9892 × 10^12^**
	Std	4.8590 × 10^23^	7.6387 × 10^33^	1.0651 × 10^33^	9.4488 × 10^32^	2.9378 × 10^24^	7.4215 × 10^16^	3.1881 × 10^16^	1.6066 × 10^38^	**7.7583 × 10^12^**
F3	Ave	4.4098 × 10^4^	5.5238 × 10^4^	1.5964 × 10^5^	9.5621 × 10^4^	6.1922 × 10^4^	2.8241 × 10^4^	5.0767 × 10^4^	5.6804 × 10^4^	**8.3114 × 10^3^**
	Std	1.0947 × 10^4^	1.7144 × 10^4^	3.7210 × 10^4^	3.5696 × 10^4^	1.1275 × 10^4^	1.1583 × 10^4^	1.1256 × 10^4^	1.2667 × 10^4^	**3.3681 × 10^3^**
F4	Ave	5.2585 × 10^2^	6.1821 × 10^2^	5.1202 × 10^2^	6.4624 × 10^2^	5.1317 × 10^2^	5.0597 × 10^2^	7.1004 × 10^2^	8.1367 × 10^3^	**4.8202 × 10^2^**
	Std	2.9692 × 10^1^	2.0992 × 10^2^	2.1201 × 10^1^	1.1306 × 10^2^	**1.7954 × 10^1^**	2.3804 × 10^1^	1.2088 × 10^2^	3.7042 × 10^3^	3.8188 × 10^1^
F5	Ave	6.4802 × 10^2^	6.5832 × 10^2^	6.9209 × 10^2^	7.3702 × 10^2^	6.9559 × 10^2^	6.2225 × 10^2^	6.9554 × 10^2^	7.8512 × 10^2^	**5.9882 × 10^2^**
	Std	3.3030 × 10^1^	3.0228 × 10^1^	1.4269 × 10^1^	5.4960 × 10^1^	**1.3094 × 10^1^**	3.4383 × 10^1^	3.2265 × 10^1^	3.4714 × 10^1^	2.9908 × 10^1^
F6	Ave	6.3982 × 10^2^	6.2087 × 10^2^	6.0086 × 10^2^	6.5289 × 10^2^	6.0195 × 10^2^	6.2937 × 10^2^	6.4985 × 10^2^	6.6317 × 10^2^	**6.0005 × 10^2^**
	Std	8.6758 × 10^0^	8.9957 × 10^0^	2.8585 × 10^-1^	1.3621 × 10^1^	6.4276 × 10^-1^	1.2878 × 10^1^	4.3994 × 10^0^	9.6443 × 10^0^	**1.2340 × 10^-1^**
F7	Ave	9.5491 × 10^2^	9.2931 × 10^2^	9.5104 × 10^2^	1.0321 × 10^3^	9.3631 × 10^2^	8.6880 × 10^2^	1.0261 × 10^3^	1.2451 × 10^3^	**8.5076 × 10^2^**
	Std	7.2265 × 10^1^	4.2149 × 10^1^	**1.6110 × 10^1^**	7.3775 × 10^1^	1.8655 × 10^1^	3.1376 × 10^1^	6.7642 × 10^1^	7.1681 × 10^1^	4.0520 × 10^1^
F8	Ave	9.2517 × 10^2^	9.2110 × 10^2^	9.9923 × 10^2^	1.0252 × 10^3^	9.8488 × 10^2^	9.1146 × 10^2^	1.0499 × 10^3^	1.0379 × 10^3^	**8.9811 × 10^2^**
	Std	2.7880 × 10^1^	2.5167 × 10^1^	**1.4565 × 10^1^**	5.4044 × 10^1^	1.5220 × 10^1^	2.6424 × 10^1^	2.6958 × 10^1^	1.8622 × 10^1^	2.4641 × 10^1^
F9	Ave	3.8477 × 10^3^	4.4491 × 10^3^	2.2953 × 10^3^	7.4125 × 10^3^	1.3123 × 10^3^	3.5631 × 10^3^	2.6024 × 10^3^	6.7736 × 10^3^	**1.1765 × 10^3^**
	Std	1.2047 × 10^3^	1.0776 × 10^3^	7.7471 × 10^2^	2.7722 × 10^3^	**2.3559 × 10^2^**	1.3125 × 10^3^	7.1535 × 10^2^	9.0925 × 10^2^	5.0249 × 10^2^
F10	Ave	4.8963 × 10^3^	4.4177 × 10^3^	7.5573 × 10^3^	6.6404 × 10^3^	7.6832 × 10^3^	4.8879 × 10^3^	**4.1716 × 10^3^**	6.5395 × 10^3^	5.0975 × 10^3^
	Std	6.2399 × 10^2^	5.1742 × 10^2^	3.0922 × 10^2^	1.1699 × 10^3^	**2.4277 × 10^2^**	6.6403 × 10^2^	5.9473 × 10^2^	4.8619 × 10^2^	1.0348 × 10^3^
F11	Ave	1.4231 × 103	1.9963 × 10^3^	2.1519 × 10^3^	1.8908 × 10^3^	1.2701 × 10^3^	1.2820 × 10^3^	1.7550 × 10^3^	2.7124 × 10^3^	**1.1985 × 10^3^**
	Std	1.2364 × 10^2^	1.0616 × 10^3^	7.7332 × 10^2^	5.6341 × 10^2^	**2.3177 × 10^1^**	5.6131 × 10^1^	1.6297 × 10^2^	1.0344 × 10^3^	5.0022 × 10^1^
F12	Ave	2.3167 × 10^7^	2.9292 × 10^7^	2.5697 × 10^7^	5.4373 × 10^7^	9.9186 × 10^5^	1.2236 × 10^7^	4.8375 × 10^5^	2.9285 × 10^9^	**1.0331 × 10^5^**
	Std	1.6452 × 10^7^	3.9613 × 10^7^	1.4775 × 10^7^	8.6360 × 10^7^	4.4788 × 10^5^	1.0567 × 10^7^	7.7607 × 10^5^	2.0293 × 10^9^	**5.8236 × 10^4^**
F13	Ave	9.6858 × 10^4^	9.0303 × 10^4^	2.6443 × 10^6^	8.7064 × 10^6^	1.8638 × 10^4^	1.1176 × 10^5^	2.7696 × 10^4^	1.3956 × 10^5^	**1.5235 × 10^4^**
	Std	4.1162 × 10^4^	4.1336 × 10^5^	4.8583 × 10^6^	1.8910 × 10^7^	**9.4613 × 10^3^**	6.1402 × 10^4^	2.5883 × 10^4^	1.3814 × 10^5^	1.2447 × 10^4^
F14	Ave	9.8899 × 10^4^	6.8194 × 10^5^	5.2854 × 10^5^	2.6880 × 10^5^	2.1858 × 10^3^	6.5224 × 10^4^	1.1323 × 10^4^	**1.6171 × 10^3^**	2.4378 × 10^3^
	Std	8.8113 × 10^4^	5.0770 × 10^5^	4.7443 × 10^5^	4.0834 × 10^5^	8.0051 × 10^2^	5.1982 × 10^4^	1.4594 × 10^4^	**6.9851 × 10^1^**	2.0571 × 10^3^
F15	Ave	5.0031 × 10^4^	6.6572 × 10^3^	3.9512 × 10^5^	1.0727 × 10^5^	5.1724 × 10^3^	5.3607 × 10^4^	9.6234 × 10^3^	9.4019 × 10^3^	**3.4686 × 10^3^**
	Std	5.2911 × 10^4^	5.1594 × 10^3^	3.2991 × 10^5^	1.0802 × 10^5^	4.2356 × 10^3^	4.3377 × 10^4^	5.3313 × 10^3^	5.8916 × 10^3^	**2.1578 × 10^3^**
F16	Ave	2.8199 × 10^3^	3.0840 × 10^3^	3.0126 × 10^3^	3.3568 × 10^3^	3.1547 × 10^3^	2.8082 × 10^3^	2.8221 × 10^3^	3.0147 × 10^3^	**2.2456 × 10^3^**
	Std	2.8000 × 10^2^	3.6801 × 10^2^	**1.9417 × 10^2^**	4.0722 × 10^2^	2.0782 × 10^2^	3.3971 × 10^2^	3.4491 × 10^2^	3.9271 × 10^2^	1.9430 × 10^2^
F17	Ave	2.1317 × 10^2^	2.3921 × 10^3^	2.2180 × 10^3^	2.6455 × 10^3^	2.0858 × 10^3^	2.1587 × 10^3^	2.6166 × 10^3^	2.1558 × 10^3^	**1.8550 × 10^3^**
	Std	2.1093 × 10^2^	2.3588 × 10^2^	1.3183 × 10^2^	2.6845 × 10^2^	1.1495 × 10^2^	2.0909 × 10^2^	2.8349 × 10^2^	1.2667 × 10^2^	**9.0259 × 10^1^**
F18	Ave	8.0407 × 10^5^	1.4113 × 10^6^	3.5970 × 10^6^	3.9830 × 10^6^	1.4249 × 10^5^	1.3471 × 10^6^	1.8414 × 10^5^	**3.5612 × 10^4^**	5.7037 × 10^4^
	Std	7.4493 × 10^5^	2.3882 × 10^6^	1.9999 × 10^6^	4.4045 × 10^6^	1.3729 × 10^5^	1.2929 × 10^6^	1.5553 × 10^5^	**2.9136 × 10^4^**	3.5610 × 10^4^
F19	Ave	2.1676 × 10^6^	9.0207 × 10^3^	5.0354 × 10^5^	6.6217 × 10^6^	5.8460 × 10^3^	5.9576 × 10^5^	1.2680 × 10^4^	1.7063 × 10^5^	**4.2762 × 10^3^**
	Std	1.4330 × 10^6^	6.9685 × 10^3^	6.7714 × 10^5^	2.0342 × 10^7^	3.2263 × 10^3^	9.0051 × 10^5^	1.0265 × 10^4^	3.0535 × 10^5^	**2.7734 × 10^3^**
F20	Ave	2.5403 × 10^3^	2.6639 × 10^3^	2.5282 × 10^3^	2.6826 × 10^3^	2.4734 × 10^3^	2.4766 × 10^3^	2.6210 × 10^3^	2.4449 × 10^3^	**2.2019 × 10^3^**
	Std	1.9672 × 10^2^	2.8078 × 10^2^	9.4061 × 10^1^	2.3210 × 10^2^	1.2799 × 10^2^	1.8194 × 10^2^	2.5989 × 10^2^	9.6247 × 10^1^	**9.2881 × 10^1^**
F21	Ave	2.4236 × 10^3^	2.4502 × 10^3^	2.4922 × 10^3^	2.5749 × 10^3^	2.4835 × 10^3^	2.4052 × 10^3^	2.5754 × 10^3^	2.5781 × 10^3^	**2.3733 × 10^3^**
	Std	3.2354 × 10^1^	3.0929 × 10^1^	**1.3765 × 10^1^**	3.8033 × 10^1^	1.7068 × 10^1^	1.8580 × 10^1^	1.9217 × 10^1^	4.7140 × 10^1^	2.0044 × 10^1^
F22	Ave	2.8635 × 10^3^	3.6435 × 10^3^	8.2993 × 10^3^	5.1692 × 10^3^	2.3087 × 10^3^	4.3348 × 10^3^	4.6175 × 10^3^	6.4418 × 10^3^	**2.3007 × 10^3^**
	Std	1.3622 × 10^3^	1.9491 × 10^3^	1.3300 × 10^3^	2.3880 × 10^3^	2.6450 × 10^0^	2.1234 × 10^3^	1.7695 × 10^3^	1.1595 × 10^3^	**1.4514 × 10^0^**
F23	Ave	2.8216 × 10^3^	3.0915 × 10^3^	2.8440 × 10^3^	3.0256 × 10^3^	2.8462 × 10^3^	2.7910 × 10^3^	2.9108 × 10^3^	3.0503 × 10^3^	**2.7211 × 10^3^**
	Std	5.8145 × 10^1^	1.3892 × 10^2^	1.8851 × 10^1^	9.2318 × 10^1^	1.8488 × 10^1^	4.6115 × 10^1^	**1.4101 × 10^1^**	6.5105 × 10^1^	2.6615 × 10^1^
F24	Ave	2.9542 × 10^3^	3.3170 × 10^3^	3.0399 × 10^3^	3.1835 × 10^3^	3.0147 × 10^3^	2.9661 × 10^3^	3.0428 × 10^3^	3.2188 × 10^3^	**2.8836 × 10^3^**
	Std	4.6096 × 10^1^	1.3003 × 10^2^	1.8516 × 10^1^	8.6162 × 10^1^	1.8863 × 10^1^	5.5403 × 10^1^	**1.2211 × 10^1^**	9.2672 × 10^1^	1.5594 × 10^1^
F25	Ave	2.9464 × 10^3^	2.9479 × 10^3^	2.9017 × 10^3^	2.9680 × 10^3^	2.9174 × 10^3^	2.9106 × 10^3^	3.1941 × 10^3^	3.9002 × 10^3^	**2.8993 × 10^3^**
	Std	2.3105 × 10^1^	3.4193 × 10^1^	**6.8458 × 10^0^**	4.7377 × 10^1^	1.7744 × 10^1^	1.7010 × 10^1^	8.8364 × 10^1^	3.5360 × 10^2^	1.7045 × 10^1^
F26	Ave	4.6539 × 10^3^	4.9468 × 10^3^	5.5982 × 10^3^	6.9814 × 10^3^	4.4188 × 10^3^	4.4033 × 10^3^	5.3116 × 10^3^	9.0990 × 10^3^	**3.3874 × 10^3^**
	Std	1.3304 × 10^3^	1.8361 × 10^3^	**1.4890 × 10^2^**	1.1188 × 10^3^	1.4182 × 10^3^	1.0682 × 10^3^	2.7459 × 10^2^	9.1218 × 10^2^	7.7003 × 10^2^
F27	Ave	3.2960 × 10^3^	3.2311 × 10^3^	3.2293 × 10^3^	3.3437 × 10^3^	3.2743 × 10^3^	3.2564 × 10^3^	3.3319 × 10^3^	3.3688 × 10^3^	**3.2231 × 10^3^**
	Std	4.5942 × 10^1^	9.4039 × 10^1^	**7.4285** × 10^0^	8.4743 × 10^1^	1.1966 × 10^1^	2.1780 × 10^1^	5.3624 × 10^1^	7.6658 × 10^1^	1.3910 × 10^1^
F28	Ave	3.3074 × 10^3^	3.3806 × 10^3^	3.3029 × 10^3^	3.4689 × 10^3^	3.2771 × 10^3^	3.2434 × 10^3^	3.6197 × 10^3^	5.5229 × 10^3^	**3.2108 × 10^3^**
	Std	2.6216 × 10^1^	1.1706 × 10^2^	1.8218 × 10^1^	2.0189 × 10^2^	2.8145 × 10^1^	2.3446 × 10^1^	1.3293 × 10^2^	6.8478 × 10^2^	**1.5174 × 10^1^**
F29	Ave	4.3235 × 10^3^	4.0141 × 10^3^	4.2032 × 10^3^	4.3755 × 10^3^	4.0285 × 10^3^	4.0544 × 10^3^	4.4109 × 10^3^	4.6288 × 10^3^	**3.5306 × 10^3^**
	Std	2.2984 × 10^2^	2.6755 × 10^2^	1.3381 × 10^2^	4.1062 × 10^2^	**1.2941 × 10^2^**	2.2522 × 10^2^	2.8289 × 10^2^	3.5041 × 10^2^	1.4579 × 10^2^
F30	Ave	7.7918 × 10^6^	8.9931 × 10^5^	4.9473 × 10^5^	2.3378 × 10^6^	1.3009 × 10^5^	3.8818 × 10^6^	1.0325 × 10^5^	2.9842 × 10^6^	**8.5852 × 10^3^**
	Std	4.8852 × 10^6^	4.7353 × 10^6^	4.7521 × 10^5^	4.8571 × 10^6^	7.1280 × 10^4^	2.5847 × 10^6^	2.2967 × 10^5^	3.2378 × 10^6^	**2.5251 × 10^3^**

**Table 3:** Experimental results of CEC2017 (dim = 50).

Function	Metric	VPPSO	IAGWO	ADE	DBO	CPO	AOO	HSO	IAO	MEIAO
F1	Ave	8.6519 × 10^8^	1.6967 × 10^7^	3.4660 × 10^8^	7.5407 × 10^9^	1.9149 × 10^8^	1.6058 × 10^6^	1.1395 × 10^5^	9.1339 × 10^10^	**3.0734 × 10^3^**
	Std	8.6138 × 10^8^	4.7766 × 10^6^	1.9634 × 10^8^	1.3188 × 10^10^	1.0618 × 10^8^	7.1646 × 10^5^	8.7181 × 10^4^	1.1713 × 10^10^	**4.2047 × 10^3^**
F2	Ave	8.2829 × 10^52^	5.9461 × 10^59^	1.8067 × 10^65^	1.2539 × 10^71^	5.6909 × 10^45^	5.1593 × 10^43^	1.8908 × 10^47^	2.4757 × 10^73^	**3.7443 × 10^30^**
	Std	3.3455 × 10^53^	1.7432 × 10^60^	7.1925 × 10^65^	6.8679 × 10^71^	1.7901 × 10^46^	2.8048 × 10^44^	8.2975 × 10^47^	1.3516 × 10^74^	**1.9272 × 10^31^**
F3	Ave	1.5443 × 10^5^	1.8438 × 10^5^	3.3024 × 10^5^	2.4440 × 10^5^	1.8671 × 10^5^	1.3498 × 10^5^	1.4124 × 10^5^	1.3800 × 10^5^	**6.2225 × 10^4^**
	Std	2.2522 × 10^4^	4.1368 × 10^4^	5.2828 × 10^4^	5.3706 × 10^4^	2.1483 × 10^4^	2.7651 × 10^4^	2.2548 × 10^4^	1.9540 × 10^4^	**1.0831 × 10^4^**
F4	Ave	8.2473 × 10^2^	7.0316 × 10^2^	7.6686 × 10^2^	1.3339 × 10^3^	7.0386 × 10^2^	6.3427 × 10^2^	1.2444 × 10^3^	2.4675 × 10^4^	**5.4567 × 10^2^**
	Std	1.1894 × 10^2^	9.8886 × 10^1^	**4.6648 × 10^1^**	4.8383 × 10^2^	6.8080 × 10^1^	5.3096 × 10^1^	2.0276 × 10^2^	4.8012 × 10^3^	5.9264 × 10^1^
F5	Ave	7.7832 × 10^2^	7.7192 × 10^2^	9.3231 × 10^2^	9.7314 × 10^2^	9.3263 × 10^2^	7.6830 × 10^2^	9.2216 × 10^2^	1.0513 × 10^3^	**7.5704 × 10^2^**
	Std	6.1473 × 10^1^	3.0960 × 10^1^	**2.0089 × 10^1^**	1.0694 × 10^2^	2.5165 × 10^1^	4.8688 × 10^1^	3.7878 × 10^1^	3.3841 × 10^1^	4.7710 × 10^1^
F6	Ave	6.5344 × 10^2^	6.3729 × 10^2^	6.0620 × 10^2^	6.6595 × 10^2^	6.1072 × 10^2^	6.5062 × 10^2^	6.6846 × 10^2^	6.8388 × 10^2^	**6.0140 × 10^2^**
	Std	7.7034 × 10^0^	7.5913 × 10^0^	1.5077 × 10^0^	9.3590 × 10^0^	2.9076 × 10^0^	1.0849 × 10^1^	3.5738 × 10^0^	5.6532 × 10^0^	**7.3146 × 10^-1^**
F7	Ave	1.2520 × 10^3^	1.1908 × 10^3^	1.2590 × 10^3^	1.4422 × 10^3^	1.2505 × 10^3^	**1.0828 × 10^3^**	1.6340 × 10^3^	1.8545 × 10^3^	1.0906 × 10^3^
	Std	1.4127 × 10^1^	7.1514 × 10^1^	**2.2955 × 10^1^**	1.4652 × 10^2^	4.1872 × 10^1^	9.4746 × 10^1^	1.8819 × 10^2^	1.0459 × 10^2^	1.0441 × 10^2^
F8	Ave	1.0802 × 10^3^	1.0818 × 10^3^	1.2327 × 10^3^	1.3194 × 10^3^	1.2184 × 10^3^	1.0689 × 10^3^	1.2888 × 10^3^	1.3764 × 10^3^	**1.0685 × 10^3^**
	Std	4.0636 × 10^1^	3.6263 × 10^1^	**2.2151 × 10^1^**	1.0216 × 10^2^	3.5062 × 10^1^	6.6929 × 10^1^	4.3488 × 10^1^	3.4139 × 10^1^	5.7494 × 10^1^
F9	Ave	1.0020 × 10^4^	1.5602 × 10^4^	8.2739 × 10^3^	2.7061 × 10^4^	8.6308 × 10^3^	1.2361 × 10^4^	9.2946 × 10^3^	2.5951 × 10^4^	**6.3306 × 10^3^**
	Std	**2.1513 × 10^3^**	3.1155 × 10^3^	2.2699 × 10^3^	7.1653 × 10^3^	2.3180 × 10^3^	4.1118 × 10^3^	2.4588 × 10^3^	2.5850 × 10^3^	6.0180 × 10^3^
F10	Ave	8.5719 × 10^3^	**7.0527 × 10^3^**	1.3867 × 10^4^	1.1596 × 10^4^	1.3639 × 10^4^	7.9258 × 10^3^	8.1840 × 10^3^	1.2242 × 10^4^	9.6841 × 10^3^
	Std	1.2876 × 10^3^	1.2947 × 10^3^	**4.2281 × 10^2^**	2.2463 × 10^3^	4.5364 × 10^2^	9.5251 × 10^2^	9.4520 × 10^2^	4.8826 × 10^2^	1.7152 × 10^3^
F11	Ave	2.5830 × 10^3^	4.8786 × 10^3^	7.8303 × 10^3^	4.3022 × 10^3^	1.8897 × 10^3^	1.5579 × 10^3^	2.8510 × 10^3^	1.5118 × 10^4^	**1.2659 × 10^3^**
	Std	5.3000 × 10^2^	3.0071 × 10^3^	2.9027 × 10^3^	1.6812 × 10^3^	2.9842 × 10^2^	8.8634 × 10^1^	8.0991 × 10^2^	3.8780 × 10^2^	**3.6211 × 10^3^**
F12	Ave	1.9132 × 10^8^	1.2752 × 10^8^	3.9502 × 10^8^	7.4820 × 10^8^	2.1047 × 10^7^	8.5109 × 10^7^	4.9589 × 10^6^	4.1808 × 10^10^	**2.8964 × 10^6^**
	Std	1.2186 × 10^8^	4.4245 × 10^8^	1.6550 × 10^8^	5.2333 × 10^8^	9.3981 × 10^6^	5.2053 × 10^7^	5.5135 × 10^6^	1.6144 × 10^10^	**1.7058 × 10^6^**
F13	Ave	1.0844 × 10^5^	5.7383 × 10^4^	6.3902 × 10^6^	1.5218 × 10^8^	1.8196 × 10^4^	1.3917 × 10^5^	3.0575 × 10^4^	8.9552 × 10^9^	**5.5717 × 10^3^**
	Std	5.5370 × 10^4^	2.0077 × 10^5^	1.0259 × 10^7^	2.2493 × 10^8^	2.6940 × 10^4^	9.0162 × 10^4^	2.0009 × 10^4^	7.2421 × 10^9^	**5.8322 × 10^3^**
F14	Ave	6.0811 × 10^5^	8.9041 × 10^6^	3.9821 × 10^6^	5.8826 × 10^6^	1.4498 × 10^5^	3.4243 × 10^5^	6.5313 × 10^4^	**3.7417 × 10^4^**	4.2656 × 10^4^
	Std	4.4206 × 10^5^	1.1458 × 10^7^	1.9004 × 10^6^	4.5658 × 10^6^	1.4222 × 10^5^	2.7049 × 10^5^	5.9888 × 10^4^	4.8767 × 10^4^	**2.9291 × 10^4^**
F15	Ave	4.0619 × 10^4^	2.1122 × 10^7^	1.1352 × 10^6^	3.8551 × 10^7^	1.3645 × 10^4^	5.3867 × 10^4^	1.4510 × 10^4^	5.3517 × 10^7^	**1.1520 × 10^4^**
	Std	2.3228 × 10^4^	6.3243 × 10^7^	1.8480 × 10^6^	9.7499 × 10^7^	6.6070 × 10^3^	3.1235 × 10^4^	**5.5899 × 10^3^**	1.3081 × 10^8^	6.0694 × 10^3^
F16	Ave	3.7966 × 10^3^	3.8958 × 10^3^	4.9647 × 10^3^	4.7601 × 10^3^	4.5602 × 10^3^	3.5355 × 10^3^	3.5296 × 10^3^	5.6611 × 10^3^	**2.8123 × 10^3^**
	Std	5.5317 × 10^2^	7.0550 × 10^2^	**2.6068 × 10^2^**	6.6489 × 10^2^	3.0039 × 10^2^	4.5633 × 10^2^	3.5069 × 10^2^	9.8291 × 10^2^	3.9447 × 10^2^
F17	Ave	3.5253 × 10^3^	3.4156 × 10^3^	3.6900 × 10^3^	4.3774 × 10^3^	3.4723 × 10^3^	3.1505 × 10^3^	3.4663 × 10^3^	3.8770 × 10^3^	**2.5950 × 10^3^**
	Std	3.8233 × 10^2^	3.3934 × 10^2^	2.0682 × 10^2^	4.9180 × 10^2^	**1.9631 × 10^2^**	3.2729 × 10^2^	2.8992 × 10^2^	5.1077 × 10^2^	2.1353 × 10^2^
F18	Ave	4.2134 × 10^6^	6.6806 × 10^6^	2.3374 × 10^7^	1.2352 × 10^7^	2.4199 × 10^6^	2.6121 × 10^6^	1.2468 × 10^6^	1.0159 × 10^6^	**4.8059 × 10^5^**
	Std	2.8302 × 10^6^	4.9074 × 10^6^	9.2913 × 10^6^	1.4539 × 10^7^	1.0963 × 10^6^	1.8049 × 10^6^	9.8430 × 10^5^	1.1779 × 10^6^	**5.1644 × 10^5^**
F19	Ave	1.2013 × 10^6^	**1.6232 × 10^4^**	6.3525 × 10^5^	8.1998 × 10^6^	1.8723 × 10^4^	1.2200 × 10^6^	2.9702 × 10^4^	1.9072 × 10^7^	1.7740 × 10^4^
	Std	1.5680 × 10^6^	8.9557 × 10^3^	9.5841 × 10^5^	1.1849 × 10^7^	**5.5918 × 10^3^**	7.6072 × 10^5^	4.6708 × 10^4^	2.7724 × 10^7^	8.1386 × 10^3^
F20	Ave	3.2165 × 10^3^	3.2702 × 10^3^	3.7755 × 10^3^	3.7526 × 10^3^	3.6186 × 10^3^	3.2645 × 10^3^	3.2091 × 10^3^	3.1639 × 10^3^	**2.7691 × 10^3^**
	Std	2.9191 × 10^2^	2.9951 × 10^2^	**1.7398 × 10^2^**	3.8260 × 10^2^	1.7512 × 10^2^	2.7182 × 10^2^	3.0907 × 10^2^	1.8463 × 10^2^	2.0685 × 10^2^
F21	Ave	2.6042 × 10^3^	2.6411 × 10^3^	2.7359 × 10^3^	2.8660 × 10^3^	2.7075 × 10^3^	2.5513 × 10^3^	2.8762 × 10^3^	2.9289 × 10^3^	**2.4772 × 10^3^**
	Std	6.4250 × 10^1^	1.0573 × 10^2^	**2.1015 × 10^1^**	6.4780 × 10^1^	2.1454 × 10^1^	4.7574 × 10^1^	3.7007 × 10^1^	7.2025 × 10^1^	5.6235 × 10^1^
F22	Ave	9.6764 × 10^3^	9.4864 × 10^3^	1.5584 × 10^4^	1.3208 × 10^4^	1.2780 × 10^4^	9.4708 × 10^3^	9.6656 × 10^3^	1.4005 × 10^4^	**7.8477 × 10^3^**
	Std	8.5974 × 10^2^	8.4512 × 10^2^	5.8690 × 10^2^	2.1911 × 10^3^	5.2676 × 10^3^	1.0315 × 10^3^	1.0172 × 10^3^	**5.6818 × 10^2^**	5.0326 × 10^3^
F23	Ave	3.0880 × 10^3^	3.8443 × 10^3^	3.1658 × 10^3^	3.5568 × 10^3^	3.1799 × 10^3^	3.0555 × 10^3^	3.2830 × 10^3^	3.7363 × 10^3^	**2.9316 × 10^3^**
	Std	7.9708 × 10^1^	4.0113 × 10^2^	**2.2225 × 10^1^**	1.3755 × 10^2^	2.6344 × 10^1^	7.0657 × 10^1^	3.7022 × 10^1^	1.8000 × 10^2^	5.9147 × 10^1^
F24	Ave	3.2494 × 10^3^	4.0540 × 10^3^	3.3516 × 10^3^	3.7382 × 10^3^	3.3427 × 10^3^	3.2264 × 10^3^	3.3456 × 10^3^	3.7743 × 10^3^	**3.0600 × 10^3^**
	Std	6.4343 × 10^1^	2.6734 × 10^2^	1.8478 × 10^1^	1.5889 × 10^2^	3.2241 × 10^1^	8.6094 × 10^1^	**1.8244 × 10^1^**	1.1511 × 10^2^	5.4740 × 10^1^
F25	Ave	3.3587 × 10^3^	3.2885 × 10^3^	3.1945 × 10^3^	3.6633 × 10^3^	3.2314 × 10^3^	3.0988 × 10^3^	3.7165 × 10^3^	1.2572 × 10^4^	**3.0974 × 10^3^**
	Std	1.1910 × 10^2^	1.0461 × 10^2^	4.7756 × 10^1^	9.3379 × 10^2^	6.4636 × 10^1^	4.1665 × 10^1^	2.4642 × 10^2^	1.6156 × 10^3^	**3.0998 × 10^1^**
F26	Ave	8.0969 × 10^3^	8.7493 × 10^3^	8.0014 × 10^3^	1.1317 × 10^4^	8.0100 × 10^3^	5.7871 × 10^3^	7.9508 × 10^3^	1.5517 × 10^4^	**3.3327 × 10^3^**
	Std	2.0458 × 10^3^	2.3274 × 10^3^	**2.3182 × 10^2^**	1.3982 × 10^3^	1.9005 × 10^3^	2.2161 × 10^3^	5.6026 × 10^2^	8.6294 × 10^2^	9.9177 × 10^2^
F27	Ave	3.7694 × 10^3^	3.7798 × 10^3^	3.5320 × 10^3^	4.0281 × 10^3^	3.7430 × 10^3^	3.6255 × 10^3^	3.7277 × 10^3^	4.1103 × 10^3^	**3.4265 × 10^3^**
	Std	1.3805 × 10^2^	8.4163 × 10^2^	8.4631 × 10^1^	3.0138 × 10^2^	9.0074 × 10^2^	1.0779 × 10^2^	1.4505 × 10^2^	2.5652 × 10^2^	**7.6224 × 10^1^**
F28	Ave	3.8533 × 10^3^	3.6211 × 10^3^	4.9503 × 10^3^	5.7094 × 10^3^	3.6853 × 10^3^	3.3839 × 10^3^	5.0837 × 10^3^	1.0372 × 10^4^	**3.3638 × 10^3^**
	Std	1.4607 × 10^2^	3.8148 × 10^2^	6.3689 × 10^2^	2.3421 × 10^3^	9.7160 × 10^1^	3.8751 × 10^1^	1.4174 × 10^3^	1.1033 × 10^3^	**2.6029 × 10^1^**
F29	Ave	5.4812 × 10^3^	4.6897 × 10^3^	5.5892 × 10^3^	6.6335 × 10^3^	5.2475 × 10^3^	5.0831 × 10^3^	5.4732 × 10^3^	8.9046 × 10^3^	**4.0101 × 10^3^**
	Std	4.0343 × 10^2^	8.8628 × 10^2^	2.9354 × 10^2^	1.1993 × 10^3^	**1.8873 × 10^2^**	4.9784 × 10^2^	3.3283 × 10^2^	1.7655 × 10^3^	3.3210 × 10^2^
F30	Ave	1.2068 × 10^8^	8.6313 × 10^7^	3.7308 × 10^7^	4.3751 × 10^7^	9.8868 × 10^6^	5.2689 × 10^7^	4.5924 × 10^6^	5.1042 × 10^8^	**9.2893 × 10^5^**
	Std	4.2359 × 10^7^	4.6330 × 10^8^	1.4515 × 10^7^	4.2792 × 10^7^	3.9091 × 10^6^	1.3199 × 10^7^	2.5314 × 10^6^	4.8616 × 10^8^	**1.6536 × 10^5^**

**Table 4:** Experimental results of CEC2017 (dim = 100).

Function	Metric	VPPSO	IAGWO	ADE	DBO	CPO	AOO	HSO	IAO	MEIAO
F1	Ave	5.6690 × 10^6^	1.7196 × 10^6^	4.7848 × 10^6^	2.5261 × 10^8^	7.8801 × 10^5^	8.4743 × 10^4^	9.1857 × 10^3^	4.1305 × 10^10^	**3.9158 × 10^3^**
	Std	5.1922 × 10^3^	4.8156 × 10^3^	5.0059 × 10^3^	1.0102 × 10^4^	4.9033 × 10^3^	3.8247 × 10^3^	6.3489 × 10^3^	2.3789 × 10^4^	**3.5421 × 10^3^**
F2	Ave	1.6098 × 10^23^	1.4519 × 10^33^	2.9671 × 10^32^	1.7732 × 10^32^	5.3667 × 10^23^	3.3146 × 10^16^	1.2694 × 10^16^	2.9482 × 10^37^	**1.9892 × 10^12^**
	Std	2.1868 × 10^4^	2.0034 × 10^4^	1.7116 × 10^4^	2.7499 × 10^4^	2.0874 × 10^4^	**1.6833 × 10^4^**	1.9302 × 10^4^	4.7235 × 10^4^	1.6872 × 10^4^
F3	Ave	4.4098 × 10^4^	5.5238 × 10^4^	1.5964 × 10^5^	9.5621 × 10^4^	6.1922 × 10^4^	2.8241 × 10^4^	5.0767 × 10^4^	5.6804 × 10^4^	**8.3114 × 10^3^**
	Std	4.3680 × 10^3^	5.5518 × 10^3^	4.2199 × 10^3^	4.7125 × 10^3^	4.2334 × 10^3^	4.0701 × 10^3^	4.2874 × 10^3^	7.0678 × 10^3^	**3.7045 × 10^3^**
F4	Ave	5.2585 × 10^2^	6.1821 × 10^2^	5.1202 × 10^2^	6.4624 × 10^2^	5.1317 × 10^2^	5.0597 × 10^2^	7.1004 × 10^2^	8.1367 × 10^3^	**4.8202 × 10^2^**
	Std	6.3830 × 10^3^	6.6003 × 10^3^	1.3796 × 10^4^	1.9015 × 10^4^	6.1584 × 10^3^	3.9625 × 10^3^	1.6191 × 10^3^	2.9617 × 10^4^	**3.7709 × 10^3^**
F5	Ave	6.4802 × 10^2^	6.5832 × 10^2^	6.9209 × 10^2^	7.3702 × 10^2^	6.9559 × 10^2^	6.2225 × 10^2^	6.9554 × 10^2^	7.8512 × 10^2^	**5.9882 × 10^2^**
	Std	1.0476 × 10^4^	8.3847 × 10^3^	1.0550 × 10^4^	1.2484 × 10^4^	1.0421 × 10^4^	8.8770 × 10^3^	9.6508 × 10^3^	7.4055 × 10^4^	**7.0258 × 10^3^**
F6	Ave	6.3982 × 10^2^	6.2087 × 10^2^	6.0086 × 10^2^	6.5289 × 10^2^	6.0195 × 10^2^	6.2937 × 10^2^	6.4985 × 10^2^	6.6317 × 10^2^	**6.0005 × 10^2^**
	Std	3.3951 × 10^8^	2.6429 × 10^7^	6.8209 × 10^6^	2.7553 × 10^8^	6.9930 × 10^6^	1.0061 × 10^8^	1.0931 × 10^6^	2.5021 × 10^10^	**2.9797 × 10^4^**
F7	Ave	1.7978 × 10^10^	3.3133 × 10^8^	7.6767 × 10^9^	9.0428 × 10^10^	1.4820 × 10^10^	4.4285 × 10^8^	3.5998 × 10^9^	2.4335 × 10^11^	**1.4937 × 10^7^**
	Std	2.9231 × 10^2^	1.7159 × 10^2^	**4.7201 × 10^1^**	2.6920 × 10^2^	9.4636 × 10^1^	1.9180 × 10^2^	6.8633 × 10^2^	8.8044 × 10^1^	2.2915 × 10^2^
F8	Ave	2.3700 × 10^137^	5.9519 × 10^147^	6.9527 × 10^154^	1.3940 × 10^154^	1.2315 × 10^127^	1.1499 × 10^121^	4.4479 × 10^203^	1.2287 × 10^162^	**3.3103 × 10^100^**
	Std	8.1577 × 10^1^	9.6108 × 10^1^	**3.5696 × 10^1^**	2.2160 × 10^2^	5.2495 × 10^1^	1.0410 × 10^2^	8.5140 × 10^1^	5.6269 × 10^1^	1.4726 × 10^2^
F9	Ave	3.7350 × 10^5^	4.4883 × 10^5^	8.9415 × 10^5^	6.1327 × 10^5^	4.5035 × 10^5^	5.6695 × 10^5^	3.6573 × 10^5^	3.0992 × 10^5^	**2.4388 × 10^5^**
	Std	8.2326 × 10^3^	8.0253 × 10^3^	9.8626 × 10^3^	1.3846 × 10^4^	8.3235 × 10^3^	6.2430 × 10^3^	1.6184 × 10^4^	**2.6347 × 10^3^**	8.6674 × 10^3^
F10	Ave	3.0095 × 10^3^	2.5116 × 10^3^	1.7042 × 10^3^	1.4390 × 10^4^	2.3712 × 10^3^	1.0543 × 10^3^	2.9120 × 10^3^	8.0259 × 10^4^	**8.9797 × 10^2^**
	Std	1.4641 × 10^3^	3.1819 × 10^3^	**6.2813 × 10^2^**	3.9617 × 10^3^	7.1984 × 10^2^	1.3901 × 10^3^	1.5560 × 10^3^	9.1512 × 10^2^	3.4596 × 10^3^
F11	Ave	1.3096 × 10^3^	1.2696 × 10^3^	1.6462 × 10^3^	1.7191 × 10^3^	1.6712 × 10^3^	1.2869 × 10^3^	1.6704 × 10^3^	1.9251 × 10^3^	**1.2139 × 10^3^**
	Std	1.4987 × 10^4^	3.0011 × 10^4^	4.9925 × 10^4^	5.0301 × 10^4^	1.0434 × 10^4^	9.5164 × 10^3^	1.9748 × 10^4^	2.5384 × 10^4^	**3.0207 × 10^3^**
F12	Ave	6.6497 × 10^2^	6.4679 × 10^2^	**6.2132 × 10^2^**	6.7982 × 10^2^	6.4497 × 10^2^	6.6323 × 10^2^	6.9137 × 10^2^	6.9736 × 10^2^	6.2753 × 10^2^
	Std	6.9959 × 10^8^	1.1215 × 10^10^	8.6264 × 10^8^	2.5333 × 10^9^	2.5200 × 10^8^	2.1755 × 10^8^	1.0898 × 10^8^	2.6480 × 10^10^	**1.3446 × 10^7^**
F13	Ave	2.5910 × 10^3^	2.2342 × 10^3^	2.2206 × 10^3^	2.9668 × 10^3^	2.3386 × 10^3^	**2.0098 × 10^3^**	4.7302 × 10^3^	3.7472 × 10^3^	2.0735 × 10^3^
	Std	5.2972 × 10^5^	8.3023 × 10^5^	5.2156 × 10^4^	3.1314 × 10^8^	1.1030 × 10^5^	2.3058 × 10^4^	3.1488 × 10^4^	9.4123 × 10^9^	**4.0211 × 10^3^**
F14	Ave	1.6638 × 10^3^	1.6255 × 10^3^	1.9413 × 10^3^	2.1508 × 10^3^	1.9688 × 10^3^	**1.5511 × 10^3^**	2.0353 × 10^3^	2.3868 × 10^3^	1.5650 × 10^3^
	Std	4.3543 × 10^6^	1.9216 × 10^7^	1.2905 × 10^7^	1.5545 × 10^7^	2.2461 × 10^6^	2.5163 × 10^6^	4.9783 × 10^5^	8.8326 × 10^6^	**3.9481 × 10^5^**
F15	Ave	**2.7534 × 10^4^**	3.9689 × 10^4^	5.1638 × 10^4^	7.2737 × 10^4^	4.6521 × 10^4^	3.5131 × 10^4^	7.0463 × 10^4^	6.1949 × 10^4^	5.0296 × 10^4^
	Std	1.8066 × 10^4^	3.4448 × 10^4^	4.1437 × 10^6^	8.7481 × 10^7^	3.6041 × 10^3^	2.3713 × 10^4^	8.3190 × 10^3^	4.9351 × 10^9^	**1.1407 × 10^3^**
F16	Ave	1.8072 × 10^4^	**1.6600 × 10^4^**	3.1780 × 10^4^	2.9696 × 10^4^	3.0286 × 10^4^	1.7317 × 10^4^	2.3907 × 10^4^	2.7702 × 10^4^	2.2987 × 10^4^
	Std	8.6796 × 10^2^	1.7218 × 10^3^	4.9578 × 10^2^	1.4118 × 10^2^	**4.3846 × 10^2^**	7.8168 × 10^2^	7.1273 × 10^2^	2.5601 × 10^3^	8.2026 × 10^2^
F17	Ave	8.0967 × 10^4^	7.8085 × 10^4^	2.2940 × 10^5^	2.1846 × 10^5^	9.1490 × 10^4^	3.2343 × 10^4^	6.1448 × 10^4^	1.3285 × 10^5^	**1.1484 × 10^4^**
	Std	4.7880 × 10^2^	1.8683 × 10^3^	3.2764 × 10^2^	1.2680 × 10^2^	**2.8908 × 10^2^**	7.0662 × 10^2^	5.3539 × 10^2^	1.0800 × 10^6^	5.5410 × 10^2^
F18	Ave	1.4614 × 10^9^	5.3044 × 10^9^	3.0583 × 10^9^	7.4353 × 10^9^	9.5192 × 10^8^	5.4585 × 10^8^	1.9414 × 10^8^	1.6006 × 10^11^	**3.1499 × 10^7^**
	Std	2.7696 × 10^6^	1.2957 × 10^7^	1.9352 × 10^7^	1.0814 × 10^7^	2.4344 × 10^6^	2.5672 × 10^6^	1.6963 × 10^6^	9.9620 × 10^6^	**8.9949 × 10^5^**
F19	Ave	1.5615 × 10^5^	2.6928 × 10^5^	6.2602 × 10^4^	3.0290 × 10^8^	1.6953 × 10^5^	7.1327 × 10^4^	7.0791 × 10^4^	3.4108 × 10^10^	**7.2444 × 10^3^**
	Std	4.3290 × 10^6^	2.0488 × 10^6^	7.0696 × 10^6^	5.1442 × 10^7^	5.8977 × 10^3^	3.6974 × 10^6^	1.6547 × 10^4^	5.9173 × 10^9^	**4.8000 × 10^3^**
F20	Ave	7.0768 × 10^6^	1.1015 × 10^7^	4.9378 × 10^7^	2.3447 × 10^7^	4.6312 × 10^6^	4.1431 × 10^6^	9.5770 × 10^5^	1.2886 × 10^7^	**8.8474 × 10^5^**
	Std	5.3028 × 10^2^	7.3502 × 10^2^	**2.3404 × 10^2^**	7.2174 × 10^2^	3.4744 × 10^2^	7.0367 × 10^2^	4.8199 × 10^2^	3.6431 × 10^2^	4.7898 × 10^2^
F21	Ave	4.9077 × 10^4^	1.7654 × 10^4^	1.9195 × 10^6^	7.2045 × 10^7^	1.0036 × 10^4^	5.6928 × 10^4^	2.7150 × 10^4^	1.2432 × 10^10^	**2.8867 × 10^3^**
	Std	1.1620 × 10^2^	6.1443 × 10^2^	3.9700 × 10^1^	1.7188 × 10^2^	**3.9176 × 10^1^**	1.5024 × 10^2^	6.6142 × 10^1^	2.1564 × 10^2^	1.6186 × 10^2^
F22	Ave	7.8501 × 10^3^	7.7264 × 10^3^	1.1560 × 10^4^	8.8447 × 10^3^	1.0407 × 10^4^	6.6807 × 10^3^	7.1982 × 10^3^	1.6782 × 10^4^	**5.6046 × 10^3^**
	Std	1.5955 × 10^3^	2.8640 × 10^3^	**5.6744 × 10^2^**	4.8342 × 10^3^	8.6736 × 10^2^	1.5331 × 10^3^	1.5333 × 10^3^	8.1040 × 10^2^	3.4484 × 10^3^
F23	Ave	5.4612 × 10^3^	5.7910 × 10^3^	8.1759 × 10^3^	8.7964 × 10^3^	6.9534 × 10^3^	5.2932 × 10^3^	5.8179 × 10^3^	4.0521 × 10^5^	**4.6531 × 10^3^**
	Std	1.6019 × 10^2^	4.9816 × 10^2^	**2.6835 × 10^1^**	2.4749 × 10^2^	5.8513 × 10^1^	1.1703 × 10^2^	5.5812 × 10^1^	2.1985 × 10^2^	1.2580 × 10^2^
F24	Ave	5.4946 × 10^6^	8.1914 × 10^6^	7.8004 × 10^7^	2.3205 × 10^7^	5.5877 × 10^6^	5.0969 × 10^6^	2.3224 × 10^6^	1.1806 × 10^7^	**1.7320 × 10^6^**
	Std	1.6784 × 10^2^	1.0848 × 10^3^	**4.3057 × 10^1^**	5.3511 × 10^2^	8.4942 × 10^1^	2.1033 × 10^2^	6.1485 × 10^1^	4.0603 × 10^2^	1.4564 × 10^2^
F25	Ave	6.1188 × 10^6^	4.0726 × 10^5^	3.7460 × 10^6^	7.4385 × 10^7^	1.2157 × 10^4^	5.6183 × 10^6^	2.2103 × 10^4^	1.3578 × 10^10^	**5.9315 × 10^3^**
	Std	3.7382 × 10^2^	9.4059 × 10^2^	4.2328 × 10^2^	6.1890 × 10^2^	2.9881 × 10^2^	1.1300 × 10^2^	6.1602 × 10^2^	2.6036 × 10^3^	**7.3012 × 10^1^**
F26	Ave	5.5012 × 10^3^	5.3500 × 10^3^	7.6557 × 10^3^	7.3139 × 10^3^	7.2926 × 10^3^	5.3243 × 10^3^	**4.8285 × 10^3^**	6.0566 × 10^3^	5.4152 × 10^3^
	Std	3.2313 × 10^3^	4.9125 × 10^3^	**4.0132 × 10^2^**	3.8208 × 10^3^	2.3566 × 10^3^	2.5457 × 10^3^	1.3165 × 10^3^	3.9457 × 10^3^	5.4463 × 10^3^
F27	Ave	3.1911 × 10^3^	4.1480 × 10^3^	3.4659 × 10^3^	4.0400 × 10^3^	3.4105 × 10^3^	3.1251 × 10^3^	3.7284 × 10^3^	4.3042 × 10^3^	**2.9183 × 10^3^**
	Std	2.5994 × 10^2^	2.4311 × 10^3^	1.7101 × 10^2^	4.2753 × 10^2^	**1.0196 × 10^2^**	1.9056 × 10^2^	2.6173 × 10^2^	7.9479 × 10^2^	1.1751 × 10^2^
F28	Ave	2.0844 × 10^4^	**2.0274 × 10^4^**	3.3890 × 10^4^	2.8096 × 10^4^	3.3137 × 10^4^	2.0285 × 10^4^	2.6034 × 10^4^	3.0667 × 10^4^	2.4171 × 10^4^
	Std	8.3988 × 10^2^	3.2609 × 10^3^	1.4842 × 10^3^	6.7571 × 10^3^	5.3220 × 10^2^	1.4525 × 10^2^	5.1755 × 10^3^	2.9447 × 10^3^	**1.0270 × 10^2^**
F29	Ave	3.9181 × 10^3^	5.9781 × 10^3^	3.8044 × 10^3^	4.8783 × 10^3^	3.9781 × 10^3^	3.7996 × 10^3^	4.0205 × 10^3^	5.0446 × 10^3^	**3.3238 × 10^3^**
	Std	8.7950 × 10^2^	3.4564 × 10^3^	**3.5979 × 10^2^**	2.6054 × 10^3^	4.2379 × 10^2^	8.6304 × 10^2^	6.7551 × 10^2^	8.7655 × 10^4^	1.0112 × 10^3^
F30	Ave	4.6572 × 10^3^	6.7949 × 10^3^	4.3381 × 10^3^	6.2359 × 10^3^	4.6052 × 10^3^	4.5040 × 10^3^	4.6155 × 10^3^	6.8111 × 10^3^	**3.9364 × 10^3^**
	Std	1.5405 × 10^8^	6.8961 × 10^7^	5.3159 × 10^6^	1.2458 × 10^8^	4.6625 × 10^6^	5.0172 × 10^7^	7.6097 × 10^5^	9.6962 × 10^9^	**1.3147 × 10^4^**

**Table 5:** Experimental results of CEC2022 (dim = 10).

Function	Metric	VPPSO	IAGWO	ADE	DBO	CPO	AOO	HSO	IAO	MEIAO
F1	Ave	3.2168 × 10^2^	3.3363 × 10^2^	5.8954 × 10^3^	1.6761 × 10^3^	4.1471 × 10^2^	3.0000 × 10^2^	1.3611 × 10^3^	5.4721 × 10^2^	**3.0000 × 10^2^**
	Std	6.2599 × 10^1^	4.2545 × 10^1^	1.7838 × 10^3^	1.5598 × 10^3^	1.0488 × 10^3^	2.5488 × 10^3^	2.7792 × 10^2^	4.7759 × 10^2^	**3.6566 × 10^-14^**
F2	Ave	4.0527 × 10^2^	4.2743 × 10^2^	4.0839 × 10^2^	4.4200 × 10^2^	**4.0050 × 10^2^**	4.1174 × 10^2^	4.5795 × 10^2^	4.1428 × 10^2^	4.1018 × 10^2^
	Std	3.9828 × 10^0^	3.4439 × 10^1^	**6.5390 × 10^-1^**	3.7532 × 10^1^	1.7707 × 10^0^	1.9706 × 10^1^	3.0717 × 10^1^	2.6762 × 10^1^	2.0850 × 10^1^
F3	Ave	6.0616 × 10^2^	6.0035 × 10^2^	6.0000 × 10^2^	6.0921 × 10^2^	6.0000 × 10^2^	6.0309 × 10^2^	6.2081 × 10^2^	6.1610 × 10^2^	**6.0000 × 10^2^**
	Std	6.0768 × 10^0^	2.7822 × 10^-1^	4.9395 × 10^-5^	6.1258 × 10^0^	2.6873 × 10^-3^	3.3009 × 10^0^	5.3945 × 10^0^	1.3370 × 10^1^	**5.9711 × 10^-14^**
F4	Ave	8.1973 × 10^2^	8.1941 × 10^2^	8.2764 × 10^2^	8.3537 × 10^2^	8.2169 × 10^2^	8.2017 × 10^2^	8.3727 × 10^2^	8.1426 × 10^2^	**8.1206 × 10^2^**
	Std	7.2116 × 10^0^	8.8887 × 10^0^	5.7921 × 10^0^	9.6418 × 10^0^	7.5712 × 10^0^	1.0092 × 10^1^	5.6815 × 10^0^	5.0031 × 10^0^	**3.9741 × 10^0^**
F5	Ave	9.1361 × 10^2^	9.2470 × 10^2^	9.0114 × 10^2^	1.0207 × 10^3^	**9.0000 × 10^2^**	9.0140 × 10^2^	9.0897 × 10^2^	1.0090 × 10^3^	9.0001 × 10^2^
	Std	1.4809 × 10^1^	4.2927 × 10^1^	1.5751 × 10^0^	1.0652 × 10^2^	**5.2372 × 10^−4^**	2.5050 × 10^0^	5.8921 × 10^0^	1.0629 × 10^2^	3.0954 × 10^-2^
F6	Ave	3.9174 × 10^3^	3.1520 × 10^3^	1.0331 × 10^4^	4.6301 × 10^3^	1.8312 × 10^3^	4.7186 × 10^3^	2.8986 × 10^3^	1.8167 × 10^3^	**1.8139 × 10^3^**
	Std	2.2285 × 10^3^	1.8163 × 10^3^	1.1309 × 10^4^	2.3130 × 10^3^	**1.3131 × 10^1^**	2.2416 × 10^3^	1.2245 × 10^3^	1.6198 × 10^1^	1.7623 × 10^1^
F7	Ave	2.0403 × 10^3^	2.0145 × 10^3^	2.0180 × 10^3^	2.0389 × 10^3^	2.0105 × 10^3^	2.0311 × 10^3^	2.0866 × 10^3^	2.0243 × 10^3^	**2.0029 × 10^3^**
	Std	1.1261 × 10^1^	9.6462 × 10^0^	2.2731 × 10^1^	1.2469 × 10^1^	**3.9909 × 10^0^**	1.3549 × 10^1^	4.0041 × 10^1^	7.1685 × 10^0^	6.8409 × 10^0^
F8	Ave	2.2250 × 10^3^	2.2214 × 10^3^	2.2208 × 10^3^	2.2273 × 10^3^	2.2182 × 10^3^	2.2245 × 10^3^	2.2729 × 10^3^	2.2161 × 10^3^	**2.2092 × 10^3^**
	Std	2.6743 × 10^0^	**1.6564 × 10^0^**	3.3783 × 10^0^	7.2016 × 10^0^	5.4018 × 10^0^	6.6343 × 10^0^	5.3377 × 10^1^	7.4940 × 10^0^	1.0120 × 10^1^
F9	Ave	2.5322 × 10^3^	**2.5235 × 10^3^**	2.5293 × 10^3^	2.5647 × 10^3^	2.5293 × 10^3^	2.5296 × 10^3^	2.6700 × 10^3^	2.5294 × 10^3^	2.5293 × 10^3^
	Std	3.9594 × 10^0^	1.3844 × 10^1^	3.5546 × 10^-9^	5.1862 × 10^1^	5.8674 × 10^-3^	9.5669 × 10^-1^	5.3833 × 10^1^	1.7476 × 10^-1^	**0.0000 × 10^0^**
F10	Ave	2.5543 × 10^3^	2.5675 × 10^3^	**2.5068 × 10^3^**	2.5465 × 10^3^	2.5079 × 10^3^	2.5717 × 10^3^	2.5906 × 10^3^	2.5207 × 10^3^	2.5431 × 10^3^
	Std	5.9795 × 10^1^	6.3640 × 10^1^	4.3278 × 10^1^	6.5626 × 10^1^	**2.8541 × 10^1^**	5.9419 × 10^1^	8.7721 × 10^1^	4.5297 × 10^1^	5.3377 × 10^1^
F11	Ave	2.7417 × 10^3^	2.7819 × 10^3^	2.6556 × 10^3^	2.7471 × 10^3^	**2.6100 × 10^3^**	2.7438 × 10^3^	2.9888 × 10^3^	2.6931 × 10^3^	2.6451 × 10^3^
	Std	1.5870 × 10^2^	1.4802 × 10^2^	8.4670 × 10^1^	1.1445 × 10^2^	**5.4772 × 10^1^**	1.6996 × 10^2^	1.7852 × 10^2^	6.0740 × 10^1^	8.0412 × 10^1^
F12	Ave	2.8639 × 10^3^	2.8761 × 10^3^	**2.8621 × 10^3^**	2.8803 × 10^3^	2.8652 × 10^3^	2.8644 × 10^3^	2.8654 × 10^3^	2.8645 × 10^3^	2.8640 × 10^3^
	Std	1.6534 × 10^0^	1.8398 × 10^1^	1.1115 × 10^0^	2.1065 × 10^1^	**6.3100 × 10^-1^**	1.6864 × 10^0^	6.6547 × 10^-1^	4.7233 × 10^0^	1.5169 × 10^0^

**Table 6:** Experimental results of CEC2022 (dim = 20).

Function	Metric	VPPSO	IAGWO	ADE	DBO	CPO	AOO	HSO	IAO	MEIAO
F1	Ave	6.5543 × 10^3^	1.0835 × 10^4^	3.8147 × 10^4^	3.2232 × 10^4^	1.2744 × 10^4^	4.3297 × 10^2^	6.8820 × 10^3^	1.4897 × 10^4^	**3.3565 × 10^2^**
	Std	1.8431 × 10^3^	5.5483 × 10^3^	8.4294 × 10^3^	9.4330 × 10^3^	2.9612 × 10^3^	1.3231 × 10^2^	2.5856 × 10^3^	5.8017 × 10^3^	**6.1709 × 10^1^**
F2	Ave	4.7581 × 10^2^	5.0561 × 10^2^	**4.4919 × 10^2^**	5.1620 × 10^2^	4.6022 × 10^2^	4.5580 × 10^2^	5.6638 × 10^2^	1.2156 × 10^3^	4.5363 × 10^2^
	Std	2.6686 × 10^1^	4.5224 × 10^1^	**8.6818 × 10^-1^**	8.5701 × 10^1^	1.0060 × 10^1^	1.4796 × 10^1^	6.8939 × 10^1^	4.3948 × 10^2^	1.0233 × 10^1^
F3	Ave	6.2761 × 10^2^	6.0829 × 10^2^	6.0008 × 10^2^	6.3927 × 10^2^	6.0028 × 10^2^	6.1992 × 10^2^	6.3864 × 10^2^	6.4093 × 10^2^	**6.0000 × 10^2^**
	Std	1.0718 × 10^1^	4.7481 × 10^0^	3.7449 × 10^-2^	1.2786 × 10^1^	1.2220 × 10^-1^	9.7333 × 10^0^	5.6199 × 10^0^	1.1421 × 10^1^	**1.2882 × 10^-2^**
F4	Ave	8.6230 × 10^2^	8.6643 × 10^2^	9.2339 × 10^2^	9.0847 × 10^2^	9.0096 × 10^2^	8.5917 × 10^2^	9.1754 × 10^2^	9.0095 × 10^2^	**8.5427 × 10^2^**
	Std	1.3695 × 10^1^	1.4935 × 10^1^	**1.0628 × 10^1^**	2.6827 × 10^1^	1.1714 × 10^1^	1.7879 × 10^1^	1.3213 × 10^1^	1.5451 × 10^1^	1.7459 × 10^1^
F5	Ave	1.5134 × 10^3^	1.9573 × 10^3^	1.2336 × 10^3^	2.3248 × 10^3^	9.1434 × 10^2^	1.5537 × 10^3^	1.0635 × 10^3^	2.3032 × 10^3^	**9.0814 × 10^2^**
	Std	3.2544 × 10^2^	3.7329 × 10^2^	2.0725 × 10^2^	6.2570 × 10^2^	**2.2688 × 10^1^**	5.9480 × 10^2^	1.2964 × 10^2^	3.0970 × 10^2^	2.5962 × 10^1^
F6	Ave	4.9920 × 10^3^	7.7143 × 10^4^	4.2204 × 10^6^	5.9065 × 10^5^	2.1873 × 10^4^	7.1835 × 10^3^	5.5208 × 10^3^	3.7837 × 10^3^	**3.4377 × 10^3^**
	Std	3.9015 × 10^3^	3.9514 × 10^5^	3.9845 × 10^6^	1.8653 × 10^6^	1.3430 × 10^4^	5.6283 × 10^3^	4.9803 × 10^3^	4.3095 × 10^3^	**2.0434 × 10^3^**
F7	Ave	2.1005 × 10^3^	2.0837 × 10^3^	2.0618 × 10^3^	2.1459 × 10^3^	2.0667 × 10^3^	2.0946 × 10^3^	2.1413 × 10^3^	2.0866 × 10^3^	**2.0359 × 10^3^**
	Std	2.8652 × 10^1^	4.9647 × 10^1^	1.0807 × 10^1^	4.7548 × 10^1^	8.8900 × 10^0^	5.1495 × 10^1^	4.1290 × 10^1^	2.5467 × 10^1^	**8.7726 × 10^0^**
F8	Ave	2.2548 × 10^3^	2.2679 × 10^3^	2.2322 × 10^3^	2.3215 × 10^3^	2.2318 × 10^3^	2.2483 × 10^3^	2.4723 × 10^3^	2.2352 × 10^3^	**2.2274 × 10^3^**
	Std	4.6085 × 10^1^	5.7438 × 10^1^	2.3511 × 10^0^	6.7093 × 10^1^	**1.7625 × 10^0^**	4.1721 × 10^1^	1.3237 × 10^2^	2.1469 × 10^1^	2.0985 × 10^0^
F9	Ave	2.5070 × 10^3^	2.4962 × 10^3^	2.4808 × 10^3^	2.5110 × 10^3^	2.4816 × 10^3^	2.4823 × 10^3^	2.7344 × 10^3^	2.5965 × 10^3^	**2.4808 × 10^3^**
	Std	1.7498 × 10^1^	1.1589 × 10^1^	2.0895 × 10^-2^	3.0914 × 10^1^	3.6710 × 10^−1^	1.9459 × 10^0^	1.3520 × 10^2^	4.2309 × 10^1^	**1.5659 × 10^-9^**
F10	Ave	3.2240 × 10^3^	2.7226 × 10^3^	2.6443 × 10^3^	3.5634 × 10^3^	**2.5322 × 10^3^**	3.3717 × 10^3^	3.6955 × 10^3^	3.4294 × 10^3^	2.5428 × 10^3^
	Std	8.6657 × 10^2^	2.5254 × 10^2^	1.8854 × 10^2^	1.2729 × 10^3^	7.1730 × 10^1^	8.8666 × 10^2^	6.5891 × 10^2^	1.2082 × 10^3^	**6.0402 × 10^1^**
F11	Ave	2.9494 × 10^3^	**2.9018 × 10^3^**	2.9103 × 10^3^	3.1460 × 10^3^	2.9152 × 10^3^	2.9463 × 10^3^	3.5900 × 10^3^	5.7796 × 10^3^	2.9333 × 10^3^
	Std	1.5379 × 10^2^	1.2195 × 10^2^	5.2174 × 10^1^	1.6539 × 10^2^	7.4140 × 10^1^	8.9500 × 10^1^	2.2898 × 10^2^	1.0570 × 10^3^	**4.7946 × 10^1^**
F12	Ave	2.9799 × 10^3^	2.9970 × 10^3^	**2.9463 × 10^3^**	3.0363 × 10^3^	2.9904 × 10^3^	2.9674 × 10^3^	3.0093 × 10^3^	3.0084 × 10^3^	2.9471 × 10^3^
	Std	3.4548 × 10^1^	1.8772 × 10^2^	**3.4108 × 10^0^**	7.2142 × 10^1^	1.1640 × 10^1^	1.9073 × 10^1^	3.6148 × 10^1^	4.5843 × 10^1^	9.0538 × 10^0^

**Table 7:** Performance outcomes different algorithms on CEC2017 and CEC2022.

Statistical Results	CEC2017 dim = 30 (+/=/−)	CEC2017 dim = 50 (+/=/−)	CEC2017 dim = 100 (+/=/−)	CEC2022 dim = 10 (+/=/−)	CEC2022 dim = 20 (+/=/−)
VPPSO	(29/0/1)	(27/0/3)	(29/0/1)	(11/0/1)	(11/0/1)
IAGWO	(28/0/2)	(23/0/7)	(27/0/3)	(9/0/3)	(11/0/1)
ADE	(30/0/0)	(30/0/0)	(28/0/2)	(11/0/1)	(11/0/1)
DBO	(28/0/2)	(30/0/0)	(30/0/0)	(12/0/0)	(12/0/0)
CPO	(29/0/1)	(28/0/2)	(30/0/0)	(11/0/1)	(12/0/0)
AOO	(28/0/2)	(24/1/5)	(27/0/3)	(11/0/1)	(11/0/1)
HSO	(30/0/0)	(25/0/5)	(26/0/4)	(12/0/0)	(12/0/0)
IAO	(29/0/1)	(30/0/0)	(30/0/0)	(9/0/3)	(11/0/1)

**Table 8:** Friedman mean rank test result.

Suites	CEC2017						CEC2022			
Dimensions	30		50		100		10		20	
Algorithms	M.R	T.R	M.R	T.R	M.R	T.R	M.R	T.R	M.R	T.R
VPPSO	4.77	4	5.03	6	4.97	4	5.08	6	4.58	5
IAGWO	4.93	5	4.23	3	3.83	3	4.83	5	4.50	4
ADE	6.10	7	6.27	7	5.67	7	4.58	4	4.33	2
DBO	7.50	9	7.47	8	7.60	8	7.75	8	7.25	8
CPO	4.00	3	4.67	4	5.23	6	3.25	2	4.42	3
AOO	3.80	2	3.37	2	3.00	2	5.33	7	4.58	5
HSO	5.53	6	4.77	5	4.97	4	8.33	9	7.25	8
IAO	7.13	8	7.80	9	8.13	9	4.42	3	6.92	7
MEIAO	**1.23**	**1**	1.40	**1**	**1.60**	**1**	**1.42**	**1**	**1.17**	**1**

**Table 9:** UAV path planning experimental results.

Algorithms	Best	Worst	Ave	Std	Runtime	Rank
VPPSO	228.5646	419.7554	320.0495	93.5191	32.04	4
IAGWO	229.0077	**379.7949**	293.8463	74.8595	37.55	5
ADE	228.5803	439.9006	329.5800	88.9737	21.15	6
DBO	228.5841	640.8841	390.1997	85.4581	**20.75**	7
CPO	228.5729	412.8294	260.8809	62.8379	21.72	2
AOO	228.6922	566.9219	404.1177	66.8448	20.91	9
HSO	228.7660	862.2279	473.9606	176.5604	21.36	8
IAO	228.5642	412.7906	341.9324	72.5110	21.10	3
MEIAO	**228.5641**	412.7769	**253.9190**	**60.6960**	21.13	**1**

## Data Availability

All data in this paper are included in this manuscript.
